# Prevalence of ultra-processed foods and beverages in newly launched products across the Americas: a comparison between the United States and Latin American countries from 2018 to 2023

**DOI:** 10.3389/fpubh.2025.1659915

**Published:** 2025-09-08

**Authors:** Gabriela Vatavuk-Serrati, Katie A. Meyer, Donna R. Miles, Lindsey Smith Taillie

**Affiliations:** ^1^Department of Nutrition, Gillings School of Global Public Health, University of North Carolina at Chapel Hill, Chapel Hill, NC, United States; ^2^Carolina Population Center, University of North Carolina at Chapel Hill, Chapel Hill, NC, United States; ^3^Nutrition Research Institute, University of North Carolina at Chapel Hill, Kannapolis, NC, United States

**Keywords:** additives, diet disparities, packaged foods and beverages, saturated fats, sodium, sugars, trends, ultra-processed foods and beverages

## Abstract

**Background:**

Ultra-processed foods (UPFs) are an increasing global health concern, but their prevalence across the food supply is unknown. This is particularly important in developing countries such as Latin America, where consumption is lower but increasing. We quantified country-specific metrics of UPFs in the food supply across the Americas, including the prevalence of UPFs, the presence and number of additives, and the extent to which UPFs and non-UPFs are high in saturated fat, sugar, and sodium (HFSS).

**Methods:**

Using data on packaged products launched between 2018 and 2023 from the Mintel Global New Products Database in 11 North and Latin American countries (*n* = 207,363 products), we identified the presence of ultra-processing markers, such as additives, in foods and beverages’ ingredient lists. We compared the prevalence of UPFs and food additives in each country to the U.S. and the mean number of additives by additive class and country. The prevalence of HFSS for ultra-processed and non-ultra-processed packaged foods and beverages was estimated in a subsample (*n* = 123,072) based on the Chilean nutrient profile model.

**Results:**

The prevalence of UPFs ranged from 69 in Venezuela to 85% in Costa Rica. Flavors and other additives were the most prevalent, ranging from 60 to 78% and 49 to 70% in Venezuela and Costa Rica, respectively. The mean number of additives ranged from 3.9 in Venezuela to 7.1 in Peru. For foods, but not beverages, a higher percentage of ultra-processed products were HFSS compared to non-ultra-processed products.

**Conclusion:**

The prevalence of UPFs among newly launched products is high across all countries in the Americas. Policies are needed to create healthier food supplies in the region.

## 1 Introduction

In recent years, public health organizations have paid close attention to ultra-processed foods (UPFs), which are industrially produced foods that contain additives and typically have high energy density and are high in saturated fats, sugar, and sodium (HFSS) (1, 2). Numerous studies have shown that consumption of UPFs is linked to increased obesity and associated health outcomes worldwide (3–15). Apart from the low-quality nutrient profile of UPFs (high in added sugars, unhealthy fats, and energy), it has been shown that ultra-processed diets are associated with the replacement of healthy dietary components, such as fiber and micronutrients (16–19). Further, some evidence suggests that UPFs are ultra-palatable and generate a reward stimulus, which has been likened to an addiction (20, 21).

Despite these health concerns, UPFs are increasingly prevalent around the globe, accounting for up to almost 60% of the average per capita daily energy intake in high-income countries (4, 5, 22–24), with rapid increases in many low- and middle-income countries (25, 26). However, significant differences remain between countries in terms of consumption, even among countries in more advanced stages of the nutrition transition. For example, recent estimates in the U.S. suggest that between 57 and 67% of total calories come from UPFs, compared to around 30% in Brazil, Chile, and Mexico (3, 27–32).

A major unresolved question relates to the prevalence of UPFs in the packaged food supply, which accounts for a sizeable proportion of diets. Understanding this is important for several reasons. First, given the flow of migrants from one country to another, understanding the prevalence of UPFs in the packaged food supply can help provide context on how individuals may be differentially exposed to UPFs over time (33–43). Second, an increasing number of countries in Latin America have implemented policies to reduce the consumption of foods HFSS. Although UPFs and HFSS are different constructs, there is a high degree of overlap between them, making it useful to understand whether countries with HFSS policies have a lower proportion of UPFs in their food supply. Lastly, alongside concerns about UPFs in general, there have been growing concerns about specific additives, including non-nutritive sweeteners (NNS), colorings, flavorings, and emulsifiers or preservatives, and their association with cancer, metabolic disturbances, and cognitive and gut issues. Despite these concerns regarding UPFs, to our knowledge, no studies have characterized the food supply in Latin American countries in terms of the prevalence of UPFs, HFSS, or additives.

To address this critical gap in the field, we leveraged a unique data resource to generate estimates of the: (1) prevalence of UPFs in the food supply of 11 countries in Latin and North America (United States, Argentina, Brazil, Chile, Colombia, Costa Rica, Ecuador, Mexico, Peru, Puerto Rico, and Venezuela) between 2018 and 2023; (2) prevalence, and average number, of additives, which include preservatives, colorants, flavor enhancers, and non-nutritive sweeteners (NNS), in the packaged food supply in the U.S. compared to Latin American countries; and (3) prevalence of foods and beverages high in calories, sugar, sodium, or saturated fat in UPFs and non-UPFs, by country.

## 2 Materials and methods

### 2.1 Study design and dataset

Data were from the Mintel Global New Products Database (GNPD), an online database of newly launched consumer products in global markets (44, 45). This database has been widely used in various studies that investigate health claims and/or nutritional content in packaged products (46–53). This study includes data from the Nutrition Facts Label (NFL) of foods and beverages available in the United States and ten Latin American countries with available data for all 6 years (Argentina, Brazil, Chile, Colombia, Costa Rica, Ecuador, Mexico, Peru, Puerto Rico, and Venezuela) from 2018 to 2023. This period of data is likely to capture what is currently on the market while still providing a large enough sample size for analysis, while using older data would have been more likely to have included products that have been discontinued or reformulated. The definition of “newly launched” encompasses products that were introduced or that underwent changes in their formulation or packaging during the study period. Secret shoppers photograph the packaging and manually enter information about the product into the Mintel system.

The data used in this study are proprietary and available via an institutional contract with Mintel. The statistical code used to generate the results is available in the Open Science Framework at DOI 10.17605/OSF. IO/PC4FX. The present study includes data on 258,513 newly launched products. Product records were excluded if they were missing the ingredients list (*n* = 8,827), had duplicate barcodes (*n* = 34,650), or included products typically classified as culinary ingredients, such as sugar and sweeteners, oils, butter, and others, or alcoholic beverages (*n* = 7,673). Alcoholic beverages were excluded from the analyses due to differences in their regulation. For instance, alcoholic beverages in the U.S., like beer, wine, and spirits, are not required to list a full ingredient list or nutritional information on their labels (54). For duplicate records, the most recent version of the product was kept. The final sample comprised 167,190 foods and 40,173 beverages.

Foods were categorized into 15 groups (baby food; bakery products; breakfast cereals; candy, gum, and chocolate; cheese and yogurt; desserts and ice cream; fruits and vegetables; other dairy products; pasta, rice, and other starchy dishes; processed fish, meat, and egg products; ready-to-eat/heat foods; sauces and seasonings; savory spreads; snacks (savory and sweet); and sweet spreads), and beverages were categorized into eight groups (beverage mixes and concentrates; carbonated soft drinks; coffee, tea, and hot chocolate; juice and fruit drinks; milk, other dairy beverages, and plant-based alternatives; meal replacements and nutritional drinks; plain and flavored water; and sports and energy drinks). Supplementary Table 1 details the foods and beverages included in each group.

### 2.2 Classification of ultra-processed foods

To identify UPFs, we adapted an algorithmic approach used by Popkin et al. (55, 56). In general, this approach identifies a product as an UPFs if it contains at least one additive that is not typically used in culinary preparations, indicating that it was processed in a factory. Popkin et al. found that the presence of additives, particularly colors and flavors, in addition to the HFSS classification, identified 100% of foods that should be targeted for healthy eating policies. We applied this approach by assessing the presence of food additives commonly utilized in UPFs, using a list based on the Codex General Standard for Food Additives (GSFA) Online Database, published by the World Health Organization (WHO) and the Food and Agriculture Organization (FAO) (57). Additives were classified according to their function into five groups: (1) flavor enhancers, (2) color additives, (3) preservatives, (4) non-nutritive sweeteners (NNS) and sugar alcohols, and (5) other additives, which include thickeners and emulsifiers, among others.

Supplementary Table 2 provides the list of additives used as search terms by class. We searched each product’s ingredient list and created variables for the presence of additives and the number of additives from each class.

### 2.3 Classification of products high in energy and nutrients of concern

To classify products “high in,” we adopted nutrient thresholds established by the final phase of the Chilean Nutrient Profile Model (NPM). The NPM is a set of guidelines that classify foods and drinks as high in nutrients of concern or energy if they contain added sugar, sodium, or saturated fats (per the ingredients list) and exceed thresholds for the total amount of these nutrients or energy on a per 100 g or per 100 mL basis for solids and liquids, respectively. We note that when applying the Chilean NPM, some products were excluded because the NPM did not apply to them (e.g., products without the addition of sugar, salt, or saturated fats) or because information about energy, sugar, sodium, or saturated fat was not available.

We chose to adopt the Chilean NPM because data on the amount of added sugar were not available from the nutrition facts panel for all products in the GNPD. In contrast, the nutrient profile model proposed by the Pan American Health Organization (PAHO) defines thresholds based on free sugars, which include added sugars as well as sugars naturally present in honey, syrups, and fruit juices and concentrates (58). Supplementary Table 3 summarizes the criteria for the classification of foods and beverages as UPFs and high in nutrients of concern and energy.

### 2.4 Data analysis

Searches for additives in the ingredient lists were performed using the *stringr* package in R. Figures were also plotted using R (version 4.4.1, R Foundation for Statistical Computing, Vienna, Austria). Statistical analyses were performed using Stata (version 18, College Station, TX). Using logit regression, we estimated country-specific proportions of products: (1) classified as UPFs, (2) containing each class of food additives, and (3) foods high in energy and HFSS. In addition, we calculated the proportion of products identified as ultra-processed by food and beverage groups. We used a Poisson regression model to estimate country-specific mean counts of additives. All analyses were corrected for multiple comparisons using Bonferroni, and a *p*-value of <0.05 was considered statistically significant.

## 3 Results

The final sample included 207,363, of which 80.6% were foods and 19.4% were beverages. The U.S. had the most newly launched products in the period between 2018 and 2023, with 81,693 products (39.4%), followed by Brazil (42,435 products, 20.5%) and Mexico (25,169, 12.1%). The country with the least newly launched products was Venezuela, with 3,030 (1.5%).

Table 1 provides information about the number of newly launched products by country. Thus, Table 1 also lists the products for which analyses of foods and beverages “high in” were limited.

**Table 1:** Total sample size of foods and beverages, by country, and products for which the Chilean NPM was applied, Mintel, 2018–2023.

Country	Total products *N* (%)	2018	2019	2020	2021	2022	2023	Complete NPM^a^ *N* (%)	% products with complete NPM^b^
United States	81,693 (39.4)	13,910	8,790	14,641	14,589	14,304	15,459	64,995 (52.8)	79.6
Argentina	12,545 (6.1)	1,904	2,102	1,971	2,201	1,967	2,400	3,452 (2.8)	27.5
Brazil	42,435 (20.5)	6,665	6,615	6,673	7,445	7,228	7,809	6,102 (5.0)	14.4
Chile	4,998 (2.4)	964	806	807	745	787	889	3,163 (2.6)	63.3
Colombia	16,999 (8.2)	2,598	2,813	2,637	2,899	2,776	3,276	12,553 (10.2)	73.8
Costa Rica	4,369 (2.1)	816	639	652	724	702	791	2,533 (2.1)	58.0
Ecuador	5,814 (2.8)	1,045	937	869	939	942	1,082	3,317 (2.7)	57.1
Mexico	25,169 (12.1)	3,647	3,503	3,679	4,608	4,657	5,075	19,126 (15.5)	76.0
Peru	5,826 (2.8)	807	948	876	1,157	998	1,040	3,473 (2.8)	59.6
Puerto Rico	4,485 (2.2)	808	699	748	881	839	510	3,542 (2.9)	79.0
Venezuela	3,030 (1.5)	484	586	417	496	517	530	816 (0.7)	26.9

a Products with added sugar, salt, or saturated fats with complete data for nutrients of concern and energy.

b The percentage was calculated based on the total number of products, by country.

**Figure 1:**
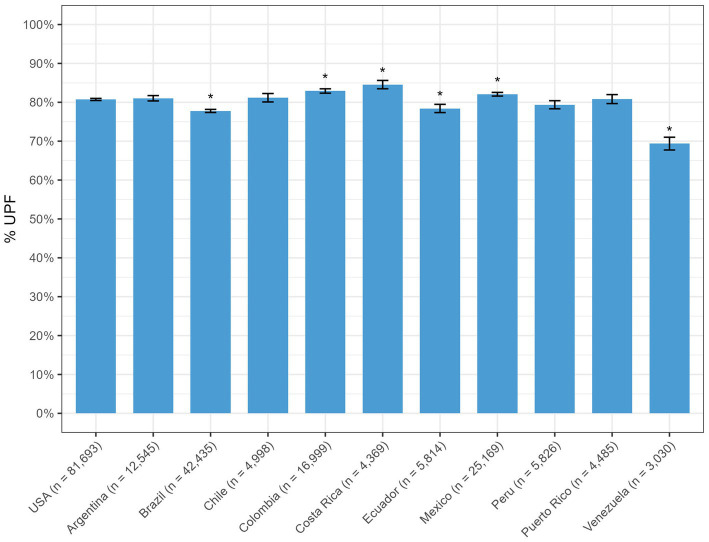
Percentage of ultra-processed foods, by country, across the U.S. and Latin America, Mintel, 2018–2023.

Figure 1 displays the percentage of newly launched products classified as ultra-processed by country, which ranged between 69 and 85% of the products in Venezuela and Costa Rica, respectively (Supplementary Table 4). Despite percentages being statistically different from the U.S., where the prevalence of UPFs was 81%, the magnitude of these differences was modest. An exception was Venezuela, where less than 70% of products were UPFs.

Overall, the percentage of beverages considered ultra-processed was slightly higher than the percentage of foods for all countries (Table 2; Supplementary Tables 5, 6). As expected, the fruits and vegetables group consistently had the lowest percentage of UPFs, ranging between 35% (Puerto Rico) and 64% (Peru). Similarly, between 25% (Venezuela) and 72% (Puerto Rico) of pasta, rice, and other starchy dishes were ultra-processed. On the other hand, candy, gum, chocolate, desserts, and ice cream had the highest prevalence of UPFs, ranging from 97% (Peru) and 99% (Chile and Colombia), and between 94% (Brazil and Ecuador) and 100% (Chile and Peru), respectively. Among beverages, ultra-processing was high for all categories, with more than 80% of beverages being UPFs across all countries. The highest percentage of UPFs observed for carbonated soft drinks and sports and energy drinks, in which almost 100% of products were UPFs.

**Table 2:** Percentage (%)^a^ of ultra-processed foods and beverages in the packaged food supply, by food categories, across the U.S. and Latin America, Mintel, 2018–2023.

	US	AR	BR	CL	CO	CR	EC	MX	PE	PR	VE
Foods											
Baby food	71	**89**	**80**	69	75	72	76	**87**	69	75	88
Bakery products	93	**94**	**80**	93	**91**	92	**88**	92	91	**97**	**77**
Breakfast cereals	81	75	**61**	**90**	78	86	85	84	**66**	80	**61**
Candy, gum, and chocolate	98	98	**95**	99	99	98	98	98	97	98	98
Cheese and yogurt	76	**98**	**96**	**98**	**93**	**91**	**96**	**92**	**99**	**83**	83
Desserts and ice cream	97	**99**	**94**	**100**	98	**99**	94	97	**100**	98	98
Fruits and vegetables	42	44	**48**	39	**56**	**55**	**56**	40	**64**	35	**55**
Other dairy products	82	77	**93**	85	**92**	90	78	**96**	**92**	79	78
Pasta, rice, and other starchy dishes	61	59	**57**	54	**45**	63	**42**	58	57	**72**	**25**
Processed fish, meat, and egg products	72	69	**77**	71	75	76	69	73	**58**	70	**31**
Ready-to-eat/heat foods	96	**85**	**62**	**83**	**78**	**88**	**79**	**89**	**86**	97	81
Sauces and seasonings	79	81	**72**	**73**	**87**	**87**	**83**	80	**88**	77	**74**
Savory spreads	80	77	77	90	87	**93**	91	87	**95**	68	75
Snacks (savory and sweet)	76	**62**	**62**	**68**	**64**	**71**	**51**	**71**	**56**	**71**	**52**
Sweet spreads	60	**78**	**77**	**84**	**89**	**87**	**79**	**82**	**86**	**89**	**93**
Total	79	**81**	**77**	**81**	**82**	**84**	**77**	**81**	78	**80**	**67**
Beverages											
Beverage mixes and concentrates	92	**99**	**99**	94	96	**97**	90	94	85	96	81
Carbonated soft drinks	98	99	99	**100**	100	**100**	**100**	96	**100**	99	97
Coffee, tea, and hot chocolate	81	**57**	**87**	75	78	81	83	80	**56**	88	**65**
Juice and fruit drinks	73	**86**	**65**	70	**87**	**88**	79	77	**96**	**84**	**97**
Milk, other dairy beverages, and plant-based alternatives	63	**78**	**91**	**80**	**88**	**91**	**81**	**77**	**92**	59	71
Meal replacements and nutritional drinks	89	**72**	88	84	90	71	84	93	**98**	93	**100**
Plain and flavored water	87	90	82	77	**73**	82	88	**80**	**63**	80	91
Sports and energy drinks	100	100	99	100	100	100	100	99	100	100	100
Total	89	**82**	**81**	**80**	**87**	89	**84**	**85**	**85**	**86**	**82**

US, United States; AR, Argentina; BR, Brazil; CL, Chile; CO, Colombia; CR, Costa Rica; EC, Ecuador; MX, Mexico; PE, Peru; PR, Puerto Rico; VE, Venezuela.

a The percentage was obtained by estimating the proportion of products classified as UPFs within each category and country.

Estimates were compared between the U.S. and other countries within food/beverage group; bolded estimates are significantly different (*p* < 0.05).

**Figure 2:**
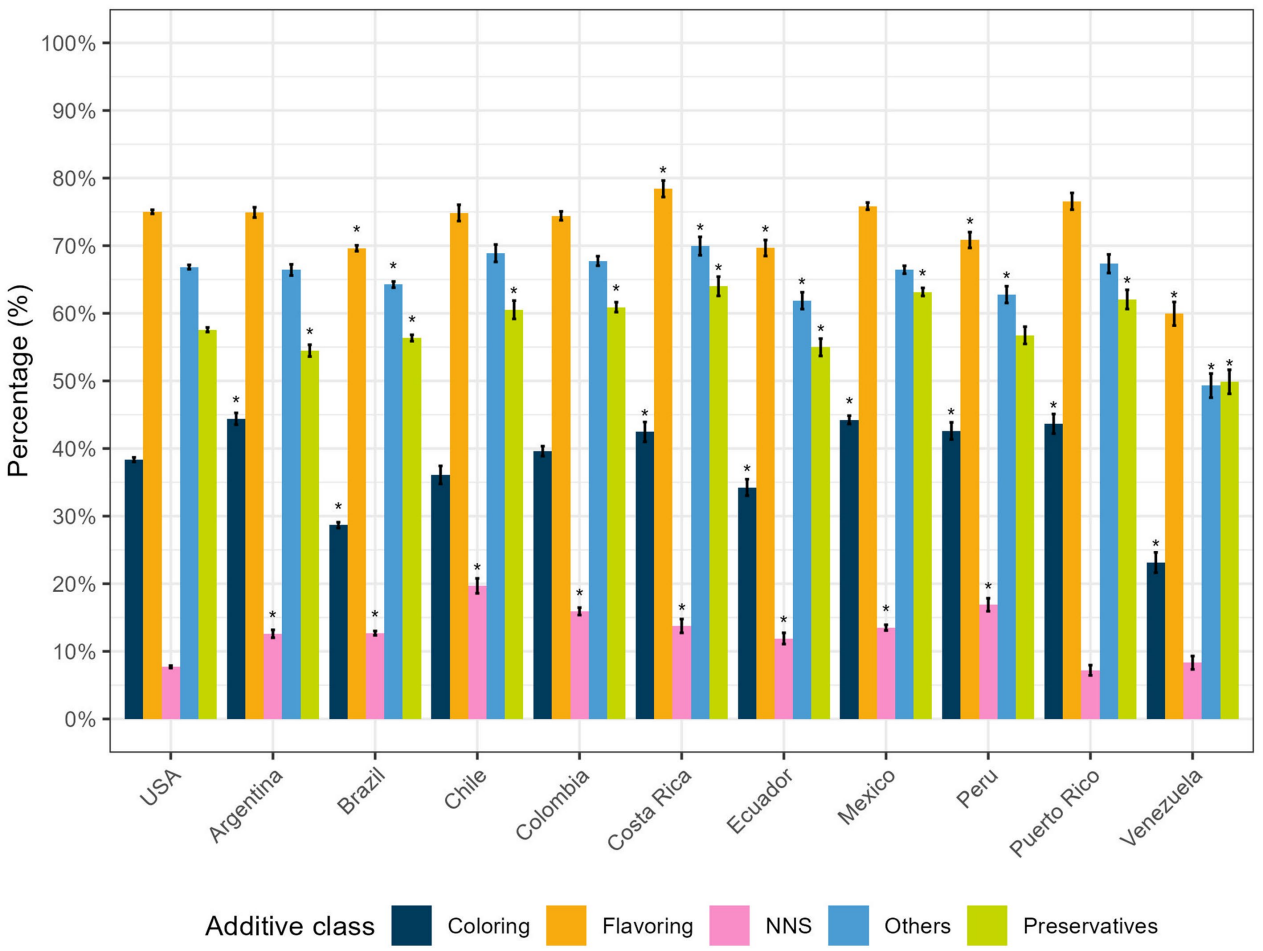
Proportion of foods and beverages containing additives across North and Latin America, by class and country, Mintel, 2018–2013.

We next examined the percentage of foods and beverages containing additives by country (Figure 2). Flavor additives were the most prevalent class in the food supply, ranging from 60% of all products in Venezuela to 78% in Costa Rica. The other additives (e.g., emulsifiers and thickeners) class was less prevalent, ranging from 49% of products in Venezuela to 70% in Costa Rica. The percentage of foods and beverages containing preservatives ranged from 50% (Venezuela) to 64% (Costa Rica). Between 23% (Venezuela) and 44% (Argentina, Mexico, and Puerto Rico) of products contained coloring. NNS were the least prevalent across all countries, ranging from 7% in Puerto Rico to 20% in Chile.

Countries varied in terms of the mean number of additives among products (Table 3). Among products that contained additives, the mean number varied between 3.9 additives for Venezuela and 7.1 for Peru. Across all countries, Venezuela had the lowest means for all categories of additives except NNS. The highest means were observed for flavor additives, which ranged from 2.8 (Venezuela) to 4.8 (U.S.), and for other additives, ranging from 2.3 (Venezuela) to 4.0 (Peru).

**Table 3:** Mean^a^ number of additives, by category, among products containing additives across North and Latin America, Mintel, 2018–2023.

Country	Any additives		Coloring		Flavoring		Preservatives		NNS^b^		Others^c^	
	Mean	*p*	Mean	*p*	Mean	*p*	Mean	*p*	Mean	*p*	Mean	*p*
United States	6.9	(Ref.)	1.7	(Ref.)	4.8	(Ref.)	2.4	(Ref.)	1.8	(Ref.)	3.9	(Ref.)
Argentina	**6.2**	<0.001	**1.9**	<0.001	**4.0**	<0.001	**2.1**	<0.001	1.8	1.000	**3.2**	<0.001
Brazil	**5.6**	<0.001	1.7	1.000	**3.8**	<0.001	**2.1**	<0.001	**1.9**	<0.001	**3.3**	<0.001
Chile	**6.7**	<0.001	**1.8**	0.002	**4.2**	<0.001	**2.2**	0.001	1.9	0.260	3.8	0.340
Colombia	**6.8**	0.007	**1.8**	<0.001	**4.6**	<0.001	**2.5**	<0.001	1.7	0.420	**3.6**	<0.001
Costa Rica	7.0	1.000	1.7	1.000	4.7	1.000	2.5	0.100	1.8	1.000	**3.8**	0.012
Ecuador	**5.5**	<0.001	**1.5**	<0.001	**3.7**	<0.001	2.3	0.304	**1.5**	<0.001	**3.1**	<0.001
Mexico	6.9	1.000	**2.0**	<0.001	**4.5**	<0.001	**2.4**	<0.001	1.7	1.000	**3.7**	<0.001
Peru	**7.1**	<0.001	**2.1**	<0.001	4.7	1.000	2.4	1.000	1.7	1.000	4.0	0.089
Puerto Rico	6.8	1.000	1.6	1.000	4.6	0.456	2.3	1.000	1.7	1.000	**3.7**	<0.001
Venezuela	**3.9**	<0.001	**1.4**	<0.001	**2.8**	<0.001	**1.7**	<0.001	1.6	1.000	**2.3**	<0.001

a Mean count among foods and beverages that contain additives.

b NNS = non-nutritive sweeteners.

c Other additives include anti-foaming agents, bulking agents, carbonating agents, emulsifiers, foaming agents, gelling agents, glazing agents, and thickeners.

Estimates were compared between the U.S. and other countries; bolded estimates are significantly different (*p* < 0.05).

**Figure 3:**
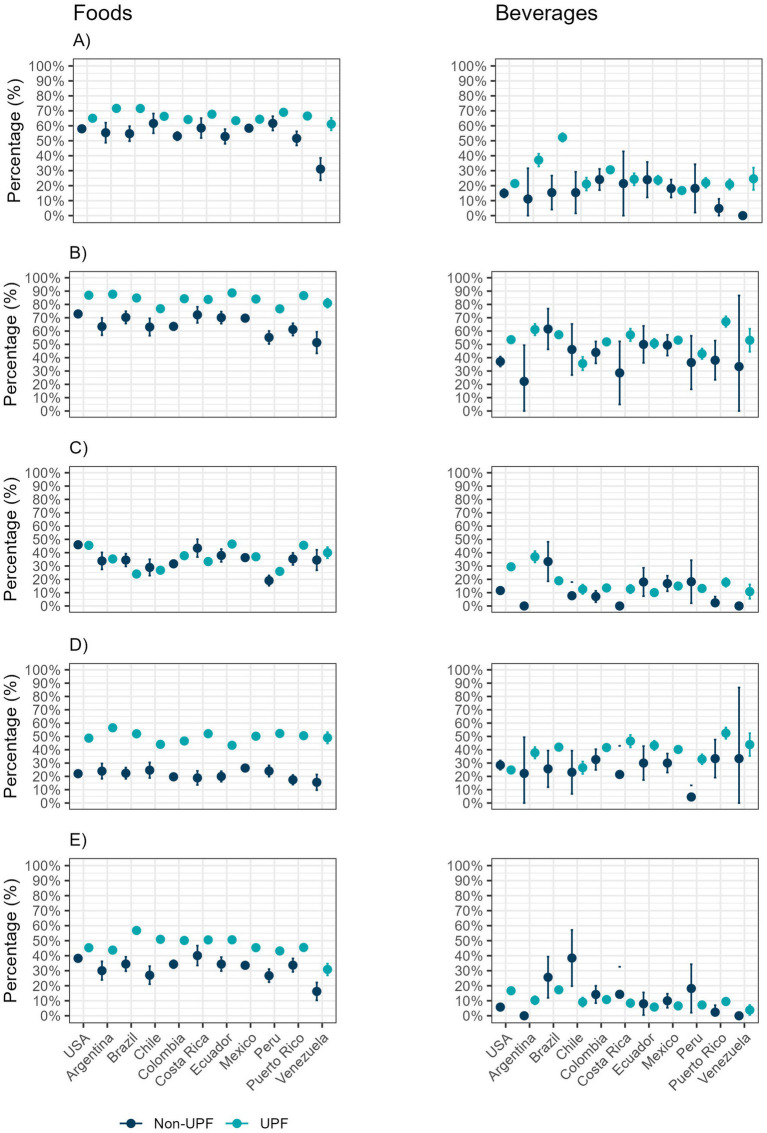
Proportion of foods and beverages “high in,” by country, 2018–2023. **(A)** High in energy; **(B)** High in any nutrient of concern; **(C)** High in sodium; **(D)** High in sugar; **(E)** High in saturated fat.

We compared the country-specific percentage of foods “high in” for UPFs and non-UPFs (Figure 3; Supplementary Table 7). As expected, a large percentage of UPFs were considered high in energy, sugar, and saturated fats. Interestingly, non-UPFs products had a similar or higher prevalence of high sodium than UPFs in most countries, except Colombia, Ecuador, Peru, and Puerto Rico.

Overall, smaller percentages of beverages were “high in” than foods (22 to 67% of beverages vs. 51–89% for foods, Figure 3; Supplementary Table 8). In Venezuela, none of the newly launched, non-UPFs beverages were classified as high in energy, sodium, or saturated fats. A higher percentage of ultra-processed beverages, compared to non-ultra-processed (17–52% vs. 0–24%), was classified as high in energy, except in Ecuador and Mexico. In general, a larger percentage of UPFs beverages were high in sugar, except in the U.S. (25–52% vs. 5–33%), and high in sodium, except in Brazil, Ecuador, Mexico, and Peru, compared to non-UPFs beverages (11–37% vs. 0–33%). In most countries, a larger prevalence of non-UPFs beverages high in saturated fats was observed, compared to UPFs, except for the U.S., Argentina, Puerto Rico, and Venezuela (0–38% vs. 4–17%).

## 4 Discussion

In this study, the prevalence of newly launched products between 2018 and 2023 in the U.S. and Latin America that were considered ultra-processed was high across all countries, with 81% in the U.S. and ranging from 69% (Venezuela) to 85% (Costa Rica). However, it is important to note that the availability of packaged foods in those countries is still different. For instance, in the U.S., over 80,000 products were introduced or modified during that period, whereas in other countries with a smaller population, such as Chile, Costa Rica, Ecuador, Peru, Puerto Rico, and Venezuela, that number was much lower, ranging between 3,000 and 6,000. The prevalence of packaged and ultra-processed foods in the diet of these countries’ populations still varies, and a high prevalence of UPFs among newly launched products does not necessarily translate into a high percentage of consumption since intake so depends on the prevalence of UPFs in existing products as well as consumption of packaged and non-packaged foods in general. Still, given the association between UPFs and adverse health outcomes (14, 59–62), the overall high prevalence of UPFs among newly launched products across countries represents an important public health concern.

The prevalence of UPFs in soft drinks was high across all countries. This finding is expected, given that sodas and sports/energy drinks typically contain colors, flavors, or sweeteners. More unexpectedly, the proportion of UPFs in the category of plain and flavored waters was also relatively high across countries (63–91%), suggesting a high prevalence of flavored waters since bottled waters are not UPFs. This high rate may potentially reflect an artifact of the Mintel database, which generates new records every time a product is reformulated or re-marketed (e.g., changes packaging). Since bottled water cannot be reformulated and may be less subject to new marketing schemes, it may have fewer records in the database than flavored waters, which is a rapidly growing category. The global flavored water market size in 2022 was estimated to be US$16.6 bi and expected to grow to US$ 40.6 billion in 10 years (63).

Mirroring the high prevalence of UPFs, a high percentage of newly launched products contained additives, particularly flavoring (60–78%) and other additives (i.e., emulsifiers, thickeners, etc.; 49–70%). Among the additives, NNS was the category with the lowest prevalence (7–20%) for all countries. Overall, Venezuela was the country with the lowest mean number of additives across all categories except NNS, likely due to the ongoing political and economic scenario (64, 65). The total number of additives varied between 3.9 (Venezuela) and 7.1 (Peru). A study investigating additives in products purchased by U.S. households found that, between 2001 and 2019, the mean number of additives increased from 4.0 to 4.6, a lower average than what was observed among newly introduced products in the same market (56). In addition, the same study found that between 49.6 and 59.5% of products contained any additive, an estimate lower than what we observed (56). This might be due to the outcomes weighted by sales volume, while the current study only focuses on product availability in the market and not on purchases by consumers. Our results were in line with a study developed in Brazil, which concluded that almost 80% of the products contained at least one additive, with flavoring agents being the most commonly found (58.8%) (66). It is possible that that percentage was even higher, given the different classifications of additive classes adopted in the study. Another study investigating the presence of 64 most commonly used additives in the U.S. estimated that 64.9% of the foods contained at least one additive (67). In France, a study revealed that 53.8% of the food supply contained at least one additive (68).

The excess of nutrients of concern and energy is one of the top reasons UPFs have drawn the attention of public health organizations. In this study, UPFs were, in general, higher in nutrients of concern and energy than non-UPFs, supporting that view, especially among foods. However, it is important to note that, although beverages had, in general, a lower percentage of products high in energy, sugar, sodium, and saturated fats than foods, they had a higher prevalence of products considered UPFs. This is because beverages often contain flavoring and coloring but are rarely considered high in nutrients of concern other than sugar. In addition, diet beverages often contain NNS, which reduces their sugar content but increases the number of additives (69–71). This indicates that, although there is an overlap between nutrient content and the presence of markers of ultra-processing, which include additives, these two approaches are not the same. This is supported by Popkin et al., who concluded that the definition of foods and beverages HFSS did not encompass all products that should be targeted by policy as unhealthy, and that the best method to identify them is a combination between Nova and HFSS (55, 72).

This study has public health implications. We found an overall equally high prevalence of UPFs in the U.S. and Latin American countries. Studies have described the transformation of the food system in Latin America in the last decades, with increased participation of large retailers and multinational food industries (73–75). The increased availability of UPFs has contributed to dietary changes, including increased UPFs intake and, consequently, multiple negative health outcomes (76–78). However, it is still unclear whether this is due to the unique aspects of processing, which can lead to overconsumption or higher energy density and, hence, higher caloric intake (79). We also found a high prevalence of food additives. However, additive categories are broad classes representing a range of different substances with different biological impacts on the body. Although studies have demonstrated that additives are considered safe in small amounts (80), others have shown associations between some of those compounds and allergic reactions, hyperactivity, gut dysbiosis, increased risk of cancer, obesity, and other metabolic disturbances, and gastrointestinal issues (81–85). It is challenging to determine the dose–response or how much individuals usually ingest, given that nutrition facts labels do not include the amount included in foods and beverages. Furthermore, most products contain multiple additives, making it difficult to pinpoint the individual impact of each additive on health (86). However, it is important to also consider the possible synergistic and antagonistic effects between different additives. For instance, a recent prospective cohort study has found associations between commonly consumed additive mixtures and type 2 diabetes (87). Future studies should focus on better understanding the potential detrimental effects of additives in the diet to inform regulations.

This study also has policy implications. During the study period, many Latin American countries have implemented food policies focusing on front-of-package warning labels to inform about the presence of nutrients of concern, such as sodium, added sugars, and saturated fats. Currently, Brazil, Chile, Colombia, Mexico, and Peru have such policies, with Mexico and Colombia also requiring warning labels for the presence of NNS and Mexico and Chile requiring a label for excess calories. This approach, while effective in informing decisions and contributing to a decrease in the intake of these nutrients, can have effects on the formulation of products since companies are incentivized to reduce sugar, sodium, and saturated fat to avoid the policy (88–93). However, these reductions in nutrients of concern do not necessarily translate to reductions in the prevalence of UPFs since sugar, sodium, and saturated fat content alone do not make a product UPFs, suggesting that such policies could keep the prevalence of UPFs stable or possibly even increase it, without improving the products’ healthfulness. An interesting example is beverages: the data from Chile show a substantial replacement of sugar with NNS in beverages, with a 35.4% increase in sweetness from NNS and a 14.5% decrease in sweetness from total sugars (94). This would seem to increase the prevalence of UPFs in beverages since NNS is an additive; however, most beverages with added sweeteners also often contain added color and/or flavor. Although the reformulation might be beneficial in reducing sugar, it is likely that the percentage of UPFs would remain the same. Unfortunately, we were unable to evaluate percentual changes in UPFs over time due to the pooled nature of our data. Annual-level data would be useful to examine how the food supply changed regarding both UPFs and HFSS in response to policy action. At a minimum, our results suggest that if policymakers want to reduce UPFs, they will need to use broader criteria than the classic approach of looking at HFSS only to address this issue.

This study is not without limitations, with the main one being related to the dataset. The GNPD includes only packaged, newly launched products. This can introduce bias to the analyses in a few different ways. First, a large proportion of minimally processed foods, such as fruits, vegetables, milk, eggs, and meats, are not packaged and thus were not included in the analyses, which can result in overestimates of the proportion of UPFs. Second, the dataset is restricted to newly launched products, and unless products underwent any reformulations and/or changes in packaging between 2018 and 2023, those were not included in the analyses despite potentially being often consumed. This could introduce selection bias since UPFs are more likely to undergo reformulations and changes in packaging as a result of marketing strategies (95–99) and thus more likely to be included in the dataset. However, despite these limitations, the study is informative about what types of products are being introduced in the markets and might reflect trends in consumer behavior and desirability.

Some other limitations include not considering the market share of products, instead attributing equal weight to every newly launched product. This prevents any inferences about the intake of UPFs, given that ultra-processed products could be consumed in smaller or larger frequency and quantity. Future studies should investigate the intersection between food supply and purchases or dietary intake regarding ultra-processing. In addition, many of the products did not contain information about nutrients of concern or energy, and this was differential across countries due to distinct local laws and regulations for the required information in the NFL and/or packaging, which limited our ability to apply the Chilean NPM and could have resulted in selection bias. In particular, our results might be an underestimation if manufacturers chose not to include that information on products that tend to be higher in those nutrients. Lastly, our algorithmic approach to identify additives and classify UPFs did not undergo a formal validity test.

The study also presents strengths. First, it investigates a large database of newly introduced foods and beverages in the U.S. and ten other markets across Latin America. Second, the database comprises information contained in the packaging of products, which results in objective information about the nutritional content. Third, identifying UPFs algorithmically proves beneficial because it allows for ingredient lists of individual products to be searched for additives, avoiding subjectivity and the classification of every product in the same group in a similar manner, despite the composition. Lastly, to identify UPFs, we searched for a comprehensive list containing more than 4,000 additives, increasing the likelihood of capturing all products.

## 5 Conclusion

Newly launched products in the U.S. and Latin America are largely ultra-processed, with a high prevalence of additives and products high in energy, sodium, sugar, and saturated fats, nutrients that contribute to the development of obesity, non-communicable diseases, and early mortality. However, since this study only reflects newly launched products, future studies should investigate the prevalence of UPFs across the entire food supply. Policies such as front-of-package labeling might be helpful in informing the general public that foods are ultra-processed.

## Data Availability

The data analyzed in this study is subject to the following licenses/restrictions: the data used in this study are proprietary and available via an institutional contract with Mintel. Requests to access these datasets should be directed to DM, drmiles@email.unc.edu.
