# Supplementary material for: Prevalence of ultra-processed foods and beverages in newly launched products across the Americas: a comparison between the United States and Latin American countries from 2018 to 2023
Source: Front Public Health. 2025 Sep 8;13:1659915. doi: 10.3389/fpubh.2025.1659915 (PMC12450670; doi:10.3389/fpubh.2025.1659915)
Supplement: Supplementary file 1 [file Table_1.docx]

Supplementary Material

**Supplementary Table 1.** Food and beverage categories

| **Foods** | |
| --- | --- |
| Baby food (n = 2,193) | Baby food, snacks, and drinks; formula for kids ages 0 to 4+ |
| Bakery products (n = 31,842) | Baking ingredients and mixes; breads; cakes; pastries; cookies; biscuits |
| Breakfast cereals (n = 5,572) | Cold and hot cereals |
| Candy, gum, and chocolate (n = 16,830) | Gum; lollipops; marshmallows; toffees; caramels; hard and soft candy; chocolates |
| Cheese and yogurt (n = 11,537) | Fresh, soft, and hard cheeses; cream cheese; dairy and plant-based spoon yogurts |
| Desserts and ice cream (n = 7,609) | Dairy-based desserts; ice cream; frozen yogurt; dessert toppings; frozen and shelf-stable desserts; sorbets; popsicles |
| Fruits and vegetables (n = 7,823) | Fresh, frozen, or canned fruits and vegetables |
| Other dairy products (n = 2,654) | Cream; coffee creamers; evaporated or condensed milk; margarine |
| Pasta, rice, and other starch dishes (n = 9,575) | Noodles; pasta; potato products; rice; polenta |
| Processed fish, meat, and egg products (n = 16,701) | Egg and egg products; fish products; meat products; meat substitutes; poultry products |
| Ready-to-eat/heat foods (n = 10,473) | Soups; instant noodles; instant rice; meal kits; prepared meals; pizzas; salads; sandwiches; wraps |
| Sauces and seasonings (n = 21,056) | Dressings; sauces; condiments; stocks; seasonings; mayonnaise; vinegar |
| Savory spreads (n = 1,994) | Dips; pates; sandwich fillers and spreads |
| Snacks - savory and sweet (n = 26,578) | Chips; popcorn; nuts; fruit snacks; cereal or energy bars |
| Sweet spreads (n = 5,226) | Caramel, chocolate, fruit or nut spreads; syrups |
| **Beverages** | |
| Beverage mixes and concentrates (n = 2,689) | Powdered and liquid concentrates for beverages |
| Carbonated soft drinks (n = 2,805) | Sodas |
| Coffee, tea, and hot chocolate (n = 4,963) | Hot or iced coffee and tea, including ready-to-drink products; malts |
| Juice and fruit drinks (n = 5,997) | Juices; nectars; fruit flavored still drinks |
| Milk, other dairy beverages, and plant-based alternatives (n = 5,873) | Plain and flavored milk; plant-based milk alternatives; drinking yogurt; other liquid dairy |
| Meal replacements and nutritional drinks (n = 3,781) | Meal replacement drinks and shakes; kombucha |
| Plain and flavored water (n = 1,829) | Water; flavored water |
| Sports and energy drinks (n = 1,763) | Electrolyte drinks; energy drinks |

**Supplementary Table 2.** Search terms used to identify food additives, by category, in the ingredient lists of packaged foods and beverages in eleven North and Latin American countries

| **Category** | **Search terms** |
| --- | --- |
| Colorants | 100, 101, 102, 103, 104, 107, 110, 120, 121, 122, 123, 124, 125, 127, 128, 129, 130, 131, 132, 133, 134, 140, 141, 142, 143, 150, 151, 152, 153, 154, 155, 160, 161, 162, 163, 164, 165, 166, 170, 171, 172, 173, 174, 175, 176, 180, 181, 182, 183, Acid Blue 9, Alkanet, Allura Red Ac, Aluminium Powder, Aluminum Powder, Amaranth, Ammonia Caramel, Annatto, Anthocyanins, Azorubine, Barley Malt Extract, Beet Red, Carotenal, Carotenic Acid, Carotene, Black Carrot Extract, Black PN, Blackcurrant Extract, Brilliant Black, Brilliant Blue FCF, Brown FK, Brown HT, Butterfly Pea Flower Extract, Calcium Carbonate, Calcium Hydrogencarbonate, Canthaxanthin, Caramel Color, Caramel Colour, Caramel I, Carbon Black, Carmine, Carmoisine, Carrot Oil, Chlorophyll, Citrus Red, Cochineal, Color, Colour, Curcumin, Enocianina, Erythrosine, Fast Green FCF, FD And C Blue, FD And C Green, FD And C Red, FD And C Yellow, Flavoxanthin, Fruit Juice Concentrate, Gardenia Blue, Gardenia Yellow, Grape Color Extract, Grape Colour Extract, Grape Skin Extract, Green S, Hibiscus Color, Hibiscus Colour, Hydrocarbon, Indigotine, Iron Oxide, Jagua Blue, Kryptoxanthin, Lithol Rubine Bk, Lutein, Lycopene, Manascorubin, Metallic Gold, Mica-Based Pearlescent Pigments, Natural Extracts, Orange B, Orchil, Paprika Extract, Paprika Oleoresin, Pas-BPP, Patent Blue V, Plain Caramel, Ponceau, Potassium Aluminium Silicate-Based Pearlescent Pigments, Purple Corn Color, Purple Corn Colour, Purple Sweet Potato Color, Purple Sweet Potato Colour, Quinoline Yellow, Red 2g, Red Cabbage Color, Red Cabbage Colour, Red Iron Oxide, Red Radish Color, Red Radish Colour, Rhodoxanthin, Riboflavin, Rubixanthin, Sandalwood, Silver, Spirulina Extract, Sulfite Caramel, Sunset Yellow FCF, Tagetes Extract, Tannic Acid, Tannins, Tartrazine, Titanium Dioxide, Toasted Partially Defatted Cooked Cottonseed Flour, Turmeric, Vegetable Carbon, Violoxanthin, Yellow 2g, Zeaxanthin, Zeaxanthis-Rich Extract From Tagetes Erecta |
| Flavoring | 1001, 101, 1100, 1101, 1104, 1-2-Butanedithiol, 133, 1-3-3-Trimethyl-2-Norbornanyl Acetate, 1-3-Butanedithiol, 1400, 1503, 1505, 1518, 1519, 1520, 1-5-5-9-Tetramethyl-13-Oxatricyclo(8.3.0.0(4-9))Tridecane, 160a, 160b, 163ii, 1-6-Hexanedithiol, 181, 1-Butanethiol, 1-Methyl-2-3-Cyclohexadione, 1-Methyl-3-Methoxy-4-Isopropylbenzene, 1-Penten-3-Ol, 1-Penten-3-One, 1-Phenyl-3 Or 5-Propylpyrazole, 1-Vinyl-3-Cyclohexenecarbaldehyde And 4-Vinyl-1-Cyclohexenecarbaldehyde, 2-(2-Butyl)-4-5-Dimethyl-3-Thiazoline, 202, 210, 211, 218, 2-2'-(Dithiodimethylene) Difuran, 220, 236, 2-3-Butanedithiol, 2-4- 3-5- And 3-6-Dimethyl-3-Cyclohexenylcarbaldehyde, 2-5 And 2-7-Dimethyl-6-7-Dihydro-5h-Cyclopentapyrazine, 250, 251, 252, 2-5-Xylenol, 260, 261i, 2-6-6-Trimethyl-1 And 2-Cyclohexen-1-Carboxaldehyde, 270, 280, 2-8-Dithianon-4-En-4-Carboxaldehyde, 290, 296, 297, 2-Acetyl-3-(5 Or 6)-Dimethylpyrazine, 2-Acetyl-3-Ethylpyrazine, 2-Acetyl-5-Methylfuran, 2-Acetylpyridine, 2-Acetylthiazole, 2-Butanol, 2-Butyl-2-Butenal, 2-Ethoxythiazol, 2-Ethyl-1-3-3-Trimethyl-2-Norbornanol, 2-Ethyl-4-Hydroxy-5-Methyl-3(2h)-Furanone, 2-Ethylpyrazine, 2-Ethylthiophenol, 2-Hexylidene Cyclopentanone, 2-Isobutyl-3-Methylpyrazine, 2-Methyl-1-3-Cyclohexadiene, 2-Methyl-1-Butanethiol, 2-Methyl-3-(2-Furyl)Acrolein, 2-Methyl-3-Furanthiol, 2-Methyl-4-Phenyl-2-Butyl Acetate, 2-Methyl-5-(Methylthio)Furan, 2-Methylheptanoic Acid, 2-Methylhexanoic Acid, 2-Methyltetrahydrothiophen-3-One, 2-Naphthalenthiol, 2-Pentenal, 2-Trans-6-Cis-Dodecadienal, 2-Trans-6-Trans-Octadienal, 2-Tridecanone, 2-Undecanol, 3-(2-Furoylthio)-2-5-Dimethylfuran, 3-(5-Methyl-2-Furyl)-Butanal, 315, 320, 321, 325, 326, 327, 330, 332, 334, 338, 3-5- And 3-6-Dimethyl-2-Isobutylpyrazine, 355, 3-5-Dimethyl-1-2-4-Trithiolane, 363, 364, 3-Acetylpyridine, 3-Benzyl-4-Heptanone, 3-Butylidenephthalide, 3-Hydroxy-5-Methyl-2-Hexanone And 2-Hydroxy-5-Methyl-3-Hexanone, 3-Methylcrotonic Acid, 3-Penten-2-One, 3-Phenylpropyl Formate, 4-(3-4-Methylenedioxyphenyl)-2-Butanone, 401, 402, 403, 404, 405, 406, 407, 410, 412, 413, 414, 416, 419, 421, 429, 432, 433, 435, 440, 444, 4-5-Dimethyl-2-Ethyl-3-Thiazoline, 460i, 461, 462, 471, 491, 4-Carvomenthenol, 4-Ethylguaiacol, 4-Hexen-1-Ol, 4-Methyl-2-Phenyl-2-Pentenal, 4-Pentenoic Acid, 504i, 508, 514i, 515i, 517, 518, 524, 530, 552, 575, 576, 578, 580, 5-Decenoic Acid, 5-Ethyl-3-Hydroxy-4-Methyl-2(5h)-Furanone, 5-Methyl-2-3-Hexanedione, 5-Methyl-3-Hexen-2-One, 5-Methylfurfural, 6-(5-Decenoyloxy)Decenoic Acid And 6-(6-Decenoyloxy)Decenoic Acid, 620, 621, 622, 623, 624, 625, 626, 627, 628, 629, 630, 631, 632, 633, 634, 635, 636, 637, 638, 639, 640, 641, 642, 650, 6-Decenoic Acid, 6-Methyl-3-5-Heptadien-2-One, 6-Methyl-5-Hepten-2-Ol, 6-Methyl-5-Hepten-2-One, 905cii, 920, 921, 942, 950, 951, 953, 955, 957, 958, 961, 968, 969, Absolute Boronia, Absolute Cassie, Absolute Civet, Acacia Gum, Acesulfame, Acetal, Acetaldehyde, Acetanisole, Acetic Acid, Acetoin, Acetonaphthone, Acetone, Acetophenone, Acetyl Butyryl, Acetyl Isobutyryl, Acetyl Nonyryl, Acetyl Propionyl, Acetyl Valeryl, Acetyl Vanillin, Acid Blue 9, Aconitic Acid, Adipic Acid, Advantame, Agar, Alanine, Aldehyde C-14, Aldehyde C-18, Alfalfa Extract, Allyl 10-Undecenoate, Allyl 2-4-Hexadienoate, Allyl 2-Ethylbutyrate, Allyl 2-Furoate, Allyl Acetic Acid, Allyl Anthranilate, Allyl Butyrate, Allyl Caproate, Allyl Cinnamate, Allyl Cyclohexane Acetate, Allyl Cyclohexane Butyrate, Allyl Cyclohexane Hexanoate, Allyl Cyclohexane Propionate, Allyl Cyclohexane Valerate, Allyl Cyclohexaneacetate, Allyl Cyclohexanebutyrate, Allyl Cyclohexanehexanoate, Allyl Cyclohexaneproprionate, Allyl Cyclohexanevalerate, Allyl Disulfide, Allyl Heptanoate, Allyl Hexanoate, Allyl Isothiocyanate, Allyl Isovalerate, Allyl Mercaptan, Allyl Methyl Disulfide, Allyl Methyl Trisulfide, Allyl Nonanoate, Allyl Octanoate, Allyl Phenoxyacetate, Allyl Phenylacetate, Allyl Propionate, Allyl Propyl Disulfide, Allyl Sorbate, Allyl Sulfide, Allyl Thiohexanoate, Allyl Thiopropionate, Allyl Tiglate, Allyl Trans- 2-Methyl-2-Butenoate, Aloe Extract, Alpha-Alpha-Dimethylbenzyl Isobutyrate, Alpha-Alpha-Dimethylphenethyl Acetate, Alpha-Alpha-Dimethylphenethyl Alcohol, Alpha-Alpha-Dimethylphenethyl Butyrate, Alpha-Alpha-Dimethylphenethyl Formate, Alpha-Amylase From Aspergillus Oryzae, Alpha-Amylase From Bacillus Subtilis, Alpha-Amylcinnamaldehyde, Alpha-Amylcinnamyl Acetate, Alpha-Amylcinnamyl Alcohol, Alpha-Amylcinnamyl Formate, Alpha-Amylcinnamyl Isovalerate, Alpha-Bisabolol, Alpha-Butylcinnamaldehyde, Alpha-Campholenic Alcohol, Alpha-Ethylbenzyl Butyrate, Alpha-Ethyl-Beta-Butylacrolein, Alpha-Ethylfuran, Alpha-Hexylcinnamaldehyde, Alpha-Irone, Alpha-Isoamyl Furfurylacetate, Alpha-Isoamyl Furfurylpropionate, Alpha-Isobutylphenethyl Alcohol, Alpha-Isomethylionyl Acetate, Alpha-Isopropyl Phenylacetaldehyde, Alpha-Ketobutyric Acid, Alpha-Methylanisylidene Acetone, Alpha-Methylbenzyl Acetate, Alpha-Methylbenzyl Alcohol, Alpha-Methylbenzyl Butyrate, Alpha-Methylbenzyl Formate, Alpha-Methylbenzyl Isobutyrate, Alpha-Methylbenzyl Propionate, Alpha-Methyl-Beta-Hydroxypropyl, Alpha-Methyl-Beta-Mercaptopropyl Sulfide, Alpha-Methylcinnamaldehyde, Alpha-Methylphenethyl Butyrate, Alpha-Phellandrene, Alpha-Phenylpropyl Butyrate, Alpha-Pinene, Alpha-Propylphenethyl Alcohol, Alpha-Santalyl Acetate, Alpha-Santalyl Phenylacetate, Alpha-Terpinene, Alpha-Terpineol, Alpha-Toluenethiol, Alpha-Toluic Acid, Alpha-Toluic Aldehyde, Ambrettolide, Ammonium Alginate, Ammonium Glutamate, Ammonium Isovalerate, Ammonium Phosphate Dibasic, Ammonium Sulfate, Ammonium Sulfide, Ammonium Sulphate, Amyl 2-Furoate, Amyl Alcohol, Amyl Butyrate, Amyl Ethyl Carbinol, Amyl Ethyl Carbinyl Acetate, Amyl Formate, Amyl Heptanoate, Amyl Hexanoate, Amyl Isothiocyanate, Amyl Methyl Disulfide, Amyl Octanoate, Amyl Vinyl Carbinol, Amylase From Aspergillus Flavus, Amylase From Aspergillus Niger, Amylase From Aspergillus Oryzae, Amylase From Bacillus Subtilis, Amylase From Bactillus Subtilis, Amylmethylacetic Acid, Angelica Lactone, Angostura Extract, Anisic Acid, Anisic Ketone, Anisole, Anisyl Acetate, Anisyl Acetone, Anisyl Alcohol, Anisyl Butyrate, Anisyl Formate, Anisyl Phenylacetate, Anisyl Propionate, Anisylmethyl Ketone, Annatto, Apiole, Arabic Gum, Asafetida Fluid Extract, Asafetida Oil, Ascorbic Acid, Asparagus Seed And Root Extract, Aspartame, Bakers Yeast Extract, Balm Oil, Barley Malt Extract, Beechwood Creosote, Benzaldehyde, Benzenethiol, Benzoic Acid, Benzoin, Benzophenone, Benzothiazole, Benzyl 2-3-Dimethylcrotonate, Benzyl 2-Methoxyethyl Acetal, Benzyl Acetate, Benzyl Acetoacetate, Benzyl Alcohol, Benzyl Benzoate, Benzyl Butyl Ether, Benzyl Butyrate, Benzyl Cinnamate, Benzyl Dipropyl Ketone, Benzyl Disulfide, Benzyl Ethyl Ether, Benzyl Formate, Benzyl Hexanoate, Benzyl Isobutyl Ketone, Benzyl Isobutyrate, Benzyl Isoeugenol, Benzyl Isothiocyanate, Benzyl Isovalerate, Benzyl Levulinate, Benzyl Mercaptan, Benzyl Methoxyethyl Acetal, Benzyl Methyl Sulfide, Benzyl Methyl Tiglate, Benzyl Nonanoate, Benzyl Phenylacetate, Benzyl Propionate, Benzyl Salicylate, Benzyl Trans-2-Methyl-2-Butenoate, Benzyldimethylcarbinyl Acetate, Benzyldimethylcarbinyl Butyrate, Benzyldimethylcarbinyl Formate, Benzylpropyl Acetate, Beta-Angelicalactone, Beta-Bourbonene, Betacarotene, Beta-Carotene, Beta-Caryophyllene, Beta-Ethylbutyl Acetate, Betaine, Beta-Ionyl Acetate, Beta-Methylphenethyl Alcohol, Beta-Naphthyl Anthranilate, Beta-Naphthyl Ethyl Ether, Beta-Naphthyl Isobutyl Ether, Beta-Naphthyl Methyl Ether, Beta-Phenylethyl Alcohol, Beta-Pinene, Beta-Santalol, Beta-Santalyl Acetate, Beta-Santalyl Phenylacetate, Beta-Terpineol, Beta-Terpinyl Anthranilate, Bha, Bht, Biphenyl, Birch Tar Oil, Bis(1-Mercaptopropyl)Sulfide, Bis(2-5-Dimethyl-3-Furyl) Disulfide, Bis(2-Methyl-3-Furyl) Disulfide, Bis(2-Methyl-3-Furyl) Disulfide, Bis(2-Methyl-3-Furyl) Tetrasulfide, Bis(2-Methylphenyl) Disulfide, Bis(Methylthio)Methane, Bisabolene, Bitterless Cascara Extract, Black Absolute Currant Buds, Black Catechu, Blackberry Bark Extract, Borneol, Bornyl Acetate, Bornyl Butyrate, Bornyl Formate, Bornyl Isovalerate, Bornyl Valerate, Brilliant Blue Fcf, Bromelain, Brown Algae Extract, Buchu Leaves, Buckbean Leaves, Butan-3-One-2-Yl Butanoate, Butanal Dibenzyl Thioacetal, Butanol, Butter Acids, Butter Esters, Butyl 10-Undecenoate, Butyl 2-Decenoate, Butyl Acetate, Butyl Acetoacetate, Butyl Alcohol, Butyl Anthranilate, Butyl Beta-(Methylthio)Acrylate, Butyl Beta-Naphthyl Ether, Butyl Butyrate, Butyl Butyryllactate, Butyl Cinnamate, Butyl Ethyl Disulfide, Butyl Ethyl Ketone, Butyl Ethyl Malonate, Butyl Formate, Butyl Heptanoate, Butyl Hexanoate, Butyl Isobutyrate, Butyl Isothiocyanate, Butyl Isovalerate, Butyl Lactate, Butyl Laurate, Butyl Levulinate, Butyl Para-Hydroxybenzoate, Butyl Phenylacetate, Butyl P-Hydroxybenzoate, Butyl Propionate, Butyl Propyl Disulfide, Butyl Salicylate, Butyl Stearate, Butyl Sulfide, Butyl Valerate, Butylamine, Butylated Hydroxyanisole, Butylated Hydroxytoluene, Butylphenylacetate, Butyraldehyde, Butyramide, Butyric Acid, Butyroin, Cadinene, Calcium 5'-Guanylate, Calcium 5'-Inosinate, Calcium 5'-Ribonucleotides, Calcium Alginate, Calcium Di-L-Glutamate, Calcium Disodium Edta, Calcium Gluconate, Calcium Glutamate, Calcium Guanylate, Calcium Lactate, Calcium Silicate, Calumba Root, Camphanol, Camphene, Campholene Acetate, Camphor, Caproic Acid, Caproic Aldehyde, Caproyl Ethanol, Caprylaldehyde, Capsicum, Carbon Dioxide, Carob Bean Gum, Carrageenan, Carvacrol, Carvacryl Ethyl Ether, Carveol, Carvone, Carvyl Acetate, Carvyl Palmitate, Carvyl Propionate, Caryophylla-3(4) 8-Dien-5-Ol (Mixture), Caryophyllene Alcohol, Cassyrane, Castor Oil, Castoreum, Cedarwood Oil, Cellulose Gel, Centaury, Cetyl Alcohol, Char Smoke Flavor, Cherry Laurel, Cherry-Laurel, Chestnut Leaves Extract, Chicory Extract, Choline Chloride, Cineole, Cinnamaldehyde, Cinnamic Acid, Cinnamyl Acetate, Cinnamyl Alcohol, Cinnamyl Benzoate, Cinnamyl Butyrate, Cinnamyl Cinnamate, Cinnamyl Formate, Cinnamyl Isobutyrate, Cinnamyl Isovalerate, Cinnamyl Phenylacetate, Cinnamyl Propionate, Cis- And Trans-1-Methoxy-1-Decene, Cis- And Trans-2-Heptylcyclopropanecarboxylic Acid, Cis- And Trans-5-Ethyl-4-Methyl-2-(1-Methylpropyl)-Thiazoline, Cis- And Trans-5-Ethyl-4-Methyl-2-(2-Methylpropyl)-Thiazoline, Cis- And Trans-Ethyl 2-4-Dimethyl-1-3-Dioxolane-2-Acetate, Cis- And Trans-Linalool Oxide, Cis- And Trans-Mercapto-P-Menthan-3-One, Cis And Trans-P-1(7)8-Menthadien-2-Yl Acetate, Cis-2-Nonen-1-Ol, Cis-2-Octenol, Cis-3 And Trans-2-Hexenyl Propionate, Cis-3-Hexen-1-Ol, Cis-3-Hexenal, Cis-3-Hexenoic Acid, Cis-3-Hexenyl Acetate, Cis-3-Hexenyl Acetoacetate, Cis-3-Hexenyl Anthranilate, Cis-3-Hexenyl Benzoate, Cis-3-Hexenyl Butyrate, Cis-3-Hexenyl Cis-3-Hexenoate, Cis-3-Hexenyl Formate, Cis-3-Hexenyl Hexanoate, Cis-3-Hexenyl Lactate, Cis-3-Hexenyl Phenylacetate, Cis-3-Hexenyl Salicylate, Cis-3-Nonen-1-Ol, Cis-3-Nonenyl Acetate, Cis-3-Octen-1-Ol, Cis-4-(2-2-3-Trimethylcyclopentyl)Butanoic Acid, Cis-4-Decenol, Cis-4-Decenyl Acetate, Cis-4-Hepten-1-Al, Cis-4-Heptenal, Cis-4-Hexenal, Cis-4-Hydroxy-6-Dodecenoic Acid Lactone, Cis-4-Octenol, Cis-5-Dodecenyl Acetate, Cis-5-Isopropenyl-Cis-2-Methylcyclopentan-1-Carboxaldehyde, Cis-5-Octen-1-Ol, Cis-5-Octenal, Cis-5-Octenoic Acid, Cis-6-Dodecenal, Cis-6-Nonen-1-Ol, Cis-6-Nonenal, Cis-6-Nonenyl Acetate, Cis-9-Dodecenal, Cis-9-Octadecenol, Cis-9-Octadecenyl Acetate, Cis-Cis-3-6-Nonadienyl Acetate, Cis-Hexenyl Isobutyrate, Cis-Hexenyl Propionate, Cis-Hexenyl Tiglate, Cis-Hexenyl Valerate, Cis-Jasmone, Citral, Citric Acid, Citronellal, Citronellol, Citronelloxyacetaldehyde, Citronellyl Acetate, Citronellyl Anthranilate, Citronellyl Butyrate, Citronellyl Formate, Citronellyl Isobutyrate, Citronellyl Phenylacetate, Citronellyl Propionate, Citronellyl Valerate, Cocoa Butter, Cocoa Extract, Coffee Extract, Collagen, Corn Syrup, Creosol, Cubebol, Cuminal, Cuminic Alcohol, Cuminyl Acetaldehyde, Cyclamen Aldehyde, Cycloheptadeca-9-En-1-One, Cyclohexaneacetic Acid, Cyclohexanecarboxylic Acid, Cyclohexaneethyl Acetate, Cyclohexanone, Cyclohexyl Acetate, Cyclohexyl Anthranilate, Cyclohexyl Butyrate, Cyclohexyl Cinnamate, Cyclohexyl Formate, Cyclohexyl Isovalerate, Cyclohexyl Propionate, Cyclopentanethiol, Cyclopentanone, Cyclopropanecarboxylic Acid (2-Isopropyl-5-Methyl-Cyclohexyl)-Amide, Cyclotene Butyrate, Cyclotene Propionate, D-2-8-P-Menthadien-1-Ol, D-3-Carene, D-8-P-Menthene-1-2-Epoxide, Damascenone, Damascone, Decalactone, Decanal, Decanoic Acid, Decenal, Decyl Acetate, Decyl Alcohol, Decyl Butyrate, Decyl Propionate, Decylic Alcohol, Dehydromenthofurolactone, Dehydronootkatone, Demineralized Whey, Dextrin, Dextrose, Di-(1-Propenyl) Sulfide, Di(Butan-3-One-1-Yl) Sulfide, Di-2-Furylmethane, Diacetyl, Diallyl Polysulfide, Diallyl Trisulfide, Dibenzyl Disulfide, Dibenzyl Ether, Dibenzyl Ketone, Dibutyl Sebacate, Dicyclohexyl Disulfide, Diethyl Acetic Acid, Diethyl Disulfide, Diethyl Malate, Diethyl Malonate, Diethyl Sebacate, Diethyl Succinate, Diethyl Sulfide, Diethyl Tartrate, Diethyl Trisulfide, Diethylacetaldehyde, Difurfuryl Ether, Digeranyl Ether, Dihydroanethole, Dihydrocarveol, Dihydrocarvyl Acetate, Dihydrocoumarin, Dihydrogalangal Acetate, Dihydromintlactone, Dihydronootkatone, Dihydroxyacetone Dimer, Dihydroxyacetophenone, Diisoamyl Disulfide, Diisoamyl Trisulfide, Diisobutyl Adipate, Diisopentyl Thiomalate, Diisopropyl Adipate, Diisopropyl Disulfide, Diisopropyl Trisulfide, Dimenthyl Glutarate, Dimercaptomethane, Dimethoxybenzene, Dimethyl Adipate, Dimethyl Anthranilate, Dimethyl Disulfide, Dimethyl Hydroquinone, Dimethyl Succinate, Dimethyl Trisulfide, Dimethyl-2-6-Octadien-1-Yl 2-Ethylbutanoate, Dimethylbenzyl Carbinol, Dimethylbenzyl Carbinyl Crotonate, Dimethylbenzyl Carbinyl Hexanoate, Dimethylphenyl Ethylcarbinyl, Dioctyl Adipate, Diphenyl Ether, Dipotassium 5'-Guanylate, Dipotassium Guanylate, Dipropyl Adipate, Dipropyl Ketone, Dipropyl Trisulfide, Di-Sec-Butyl Disulfide, Disodium 5'-Guanylate, Disodium 5'-Inosinate, Disodium 5'-Ribonucleotides, Disodium Guanylate, Disodium Inosinate, Disodium Succinate, Divanillin, Dl-(3-Amino-3-Carboxypropyl)Dimethylsulfonium Chloride, Dl-Cystine, D-Limonen-10-Ol, D-Limonene, Dl-Isoleucine, Dl-Isomenthol, Dl-Isomenthone, Dl-Menthone 1-2-Glycerol Ketal, Dl-Methionine, Dl-Phenylalanine, Dl-Valine, D-Neo-Menthol, Dodecalactone, Dodecanal, Dodecanethiol, Dodecenal, Dodecyl Butyrate, Dodecyl Isobutyrate, Dodecyl Lactate, Dodecyl Propionate, Dog Grass Extract, Dried Algae Meal, Dried Yeasts, Elder Flowers Extract, Elemi Gum, Elemi Oil, Elemicin, Enanthaldehyde, Enanthic Alcohol, Enzyme-Modified Whole Milk Powder, Epoxyoxophorone, Erythorbic Acid, Erythritol, Erythro- And Threo-3-Mercapto-2-Methylbutan-1-Ol, Estragole, Ethane-1-1-Dithiol, Ethanethiol, Ethanol, Ethone, Ethoxybenzaldehyde, Ethyl (P-Tolyloxy)Acetate, Ethyl 10-Undecenoate, Ethyl 2-(Methyldithio)Propionate, Ethyl 2-(Methylthio)Acetate, Ethyl 2-4-7-Decatrienoate, Ethyl 2-4-Dioxohexanoate, Ethyl 2-4-Hexadienoate, Ethyl 2-5-Dimethyl-3-Oxo-4(2h)-Furyl Carbonate, Ethyl 2-Acetyl-3-Phenylpropionate, Ethyl 2-Acetylhexanoate, Ethyl 2-Acetyloctanoate, Ethyl 2-Ethyl-3-Phenylpropanoate, Ethyl 2-Ethylbutyrate, Ethyl 2-Ethylhexanoate, Ethyl 2-Furanpropionate, Ethyl 2-Hexenoate (Mixture Of Isomers), Ethyl 2-Hydroxy-3-Phenylpropionate, Ethyl 2-Hydroxyethyl Sulfide, Ethyl 2-Mercapto-2-Methylpropionate, Ethyl 2-Mercaptopropionate, Ethyl 2-Methyl Pentanoate, Ethyl 2-Methyl-3-4-Pentadienoate, Ethyl 2-Methyl-3-Pentenoate, Ethyl 2-Methylbutyrate, Ethyl 2-Nonynoate, Ethyl 3-(2-Furyl)Propanoate, Ethyl 3-(2-Hydroxyphenyl)Propanoate, Ethyl 3-(Ethylthio)Butyrate, Ethyl 3-(Furfurylthio)Propionate, Ethyl 3-(Methylthio)-(2e)-Propenoate, Ethyl 3-(Methylthio)-(2z)-Propenoate, Ethyl 3-(Methylthio)-2-Propenoate, Ethyl 3-(Methylthio)Butyrate, Ethyl 3-Acetoxy-2-Methylbutyrate, Ethyl 3-Hexenoate, Ethyl 3-Hydroxybutyrate, Ethyl 3-Hydroxyhexanoate, Ethyl 3-Hydroxyoctanoate, Ethyl 3-Mercaptopropionate, Ethyl 3-Methyl-2-Oxopentanoate, Ethyl 3-Methylpentanoate, Ethyl 3-Methylthiopropionate, Ethyl 3-Octenoate, Ethyl 3-Oxohexanoate, Ethyl 3-Phenylglycidate, Ethyl 3-Phenylpropionate, Ethyl 4-(Acetylthio)Butyrate, Ethyl 4-(Methylthio)Butyrate, Ethyl 4-Methylpentanoate, Ethyl 4-Pentenoate, Ethyl 4-Phenylbutyrate, Ethyl 5-(Methylthio)Valerate, Ethyl 5-Acetoxyoctanoate, Ethyl 5-Formyloxydecanoate, Ethyl 5-Hexenoate, Ethyl 5-Hydroxydecanoate, Ethyl 5-Hydroxyoctanoate, Ethyl 5-Oxodecanoate, Ethyl Acetate, Ethyl Acetoacetate, Ethyl Aconitate, Ethyl Acrylate, Ethyl Alcohol, Ethyl Alpha-Acetylcinnamate, Ethyl Alpha-Ethyl-Beta-Methyl-Beta-Phenylglycidate, Ethyl Amyl Ketone, Ethyl Anthranilate, Ethyl Benzoate, Ethyl Benzoylacetate, Ethyl Brassylate, Ethyl Butyl Carbinol, Ethyl Butyl Ketone, Ethyl Butyrate, Ethyl Cellulose, Ethyl Cinnamate, Ethyl Cis-4-7-Octadienoate, Ethyl Cis-4-Heptenoate, Ethyl Cis-4-Octenoate, Ethyl Crotonate, Ethyl Cyclohexanecarboxylate, Ethyl Cyclohexanepropionate, Ethyl Decanoate, Ethyl Dodecanoate, Ethyl Formate, Ethyl Furfuryl Ether, Ethyl Heptanoate, Ethyl Hexadecanoate, Ethyl Hexanoate, Ethyl Hydrocinnamate, Ethyl Isobutyrate, Ethyl Isoeugenol, Ethyl Isothiocyanate, Ethyl Isovalerate, Ethyl Lactate, Ethyl Laurate, Ethyl Levulinate, Ethyl Linalyl Ether, Ethyl Malonate, Ethyl Maltol, Ethyl Methyl Disulfide, Ethyl Methylphenylglycidate, Ethyl Methyl-P-Tolylglycidate, Ethyl Myristate, Ethyl N-Ethylanthranilate, Ethyl Nitrite, Ethyl N-Methylanthranilate, Ethyl Nonanoate, Ethyl Octadecanoate, Ethyl Octanoate, Ethyl Octyne Carbonate, Ethyl Oleate, Ethyl Oxyhydrate, Ethyl P-Anisate, Ethyl Pentanoate, Ethyl Phenylacetate, Ethyl Propionate, Ethyl Propyl Disulfide, Ethyl Propyl Trisulfide, Ethyl Pyruvate, Ethyl Salicylate, Ethyl Sorbate, Ethyl Tetradecanoate, Ethyl Thioacetate, Ethyl Tiglate, Ethyl Trans-2-Butenoate, Ethyl Trans-2-Cis-4-Decadienoate, Ethyl Trans-2-Decenoate, Ethyl Trans-2-Hexenoate, Ethyl Trans-2-Methyl-2-Butenoate, Ethyl Trans-2-Octenoate, Ethyl Trans-4-Decenoate, Ethyl Undecanoate, Ethyl Valerate, Ethyl Vanillin, Ethyl-(3 5 Or 6)-Methoxypyrazine (85%) And 2-Methyl-(3 5 Or 6)-Methoxypyrazine (13%), Ethyl-2-(4-Hydroxy-3-Methoxy-Phenyl)Acetate, Ethyl-2-Methyl-2-Pentenoate, Ethyl-3-Hexenoate, Ethylamine, Ethylbenzyl Acetoacetate, Ethylcellulose, Ethylcyclo-Pentenolone, Ethylene Brassylate, Eucalyptol, Eugenol, Eugenyl Acetate, Eugenyl Benzoate, Eugenyl Formate, Eugenyl Isovalerate, Farnesene, Farnesol, Fd And C Blue, Fd And C Red, Fd And C Yellow, Fenchone, Fenchyl Alcohol, Ficin, Flavor, Flavour, Formic Acid, Fumaric Acid, Furfural, Furfuryl 2-Methyl-3-Furyl Disulfide, Furfuryl 3-Methylbutanoate, Furfuryl Acetate, Furfuryl Alcohol, Furfuryl Butyrate, Furfuryl Decanoate, Furfuryl Formate, Furfuryl Isopropyl Sulfide, Furfuryl Mercaptan, Furfuryl Methyl Ether, Furfuryl Methyl Sulfide, Furfuryl Octanoate, Furfuryl Pentanoate, Furfuryl Propionate, Furyl Acetone, Fusel Oil Refined, Galangal Root, Galbanum, Gambir, Gamma-Heptalactone, Gamma-Hexalactone, Gamma-Methyldecalactone, Gamma-Nonalactone, Gamma-Octadecalactone, Gamma-Octalactone, Gamma-Undecalactone, Gamma-Valerolactone, Geraniol, Geranyl Acetate, Geranyl Acetoacetate, Geranyl Acetone, Geranyl Benzoate, Geranyl Butyrate, Geranyl Formate, Geranyl Hexanoate, Geranyl Isobutyrate, Geranyl Isovalerate, Geranyl Phenylacetate, Geranyl Propionate, Ghatti Gum, Glucono Delta-Lactone, Glucose Pentaacetate, Glutamate, Glutamic Acid, Glutamyl-2-Aminobutyric Acid, Glutamyl-Norvaline, Glutamyl-Norvalyl-Glycine, Glutamyl-Valyl-Glycine, Glycerol 5-Hydroxydecanoate, Glycerol 5-Hydroxydodecanoate, Glyceryl Monooleate, Glyceryl Monostearate, Glyceryl Tribenzoate, Glyceryl Tripropanoate, Glycine, Glycyrrhizin, Grape Skin Extract, Guaiacol, Guaiacyl Acetate, Guaiacyl Phenylacetate, Guaiene, Guaiol Acetate, Guanylic Acid, Guar, Guarana Gum, Gum Arabic, Gum Ghatti, Gum Tragacanth, Heliotropyl Acetate, Heptanal, Heptane-1-Thiol, Heptanoic Acid, Hept-Trans-2-En-1-Yl Acetate, Heptyl Acetate, Heptyl Alcohol, Heptyl Butyrate, Heptyl Cinnamate, Heptyl Formate, Heptyl Heptanoate, Heptyl Isobutyrate, Heptyl Octanoate, Heptylidene Acetone, Hesperidin, Hexadecyl Lactate, Hexalactone, Hexanal, Hexanoic Acid, Hexyl 2-Butenoate, Hexyl 2-Furoate, Hexyl 2-Methyl-3 And 4-Pentenoate (Mixture), Hexyl 2-Methylbutanoate, Hexyl 2-Methylbutyrate, Hexyl 3-Mercaptobutanoate, Hexyl 3-Methylbutanoate, Hexyl Acetate, Hexyl Alcohol, Hexyl Benzoate, Hexyl Butyrate, Hexyl Decanoate, Hexyl Formate, Hexyl Heptanoate, Hexyl Hexanoate, Hexyl Isobutyrate, Hexyl Isothiocyanate, Hexyl Isovalerate, Hexyl Nonanoate, Hexyl Octanoate, Hexyl Phenylacetate, Hexyl Propionate, Hexyl Trans-2-Hexenoate, Hexylamine, Hexylmethylcarbinol, High Fructose Corn Syrup, High-Fructose Corn Syrup, Homoeriodictyol Sodium Salt, Homoguaiacol, Hvp, Hydratropaldehyde, Hydratropic Aldehyde, Hydratropyl Alcohol, Hydratropyl Butyrate, Hydratropyl Isobutyrate, Hydrocinnamaldehyde, Hydrocinnamic Acid, Hydrocinnamyl Acetate, Hydrocinnamyl Alcohol, Hydrocinnamyl Cinnamate, Hydrocinnamyl Formate, Hydrocinnamyl Hexanoate, Hydrocinnamyl Isobutyrate, Hydrocinnamyl Isovalerate, Hydrogenated Isomaltulose Isomaltitol, Hydrogenated Soybean Oil, Hydrogenated Tallow, Hydrolysed Animal Protein, Hydrolysed Protein, Hydrolysed Vegetable Protein, Hydrolyzed Animal Protein, Hydrolyzed Protein, Hydrolyzed Vegetable Protein, Hydroquinone Monoethyl Ether, Hydroxy(4-Hydroxy-3-Methoxyphenyl)Acetic Acid, Hydroxyacetone, Hydroxycitronellal, Hydroxynonanoic Acid Delta-Lactone, Hydroxypropyl Alginate, Indole, Inosinic Acid, Invert Sugar, Ionol, Ionone, Isoambrettolide, Isoamyl 2-Furanbutyrate, Isoamyl 2-Furanpropionate, Isoamyl 2-Methylbutyrate, Isoamyl 3-(2-Furan)Propionate, Isoamyl 4-(2-Furan)Butyrate, Isoamyl Acetate, Isoamyl Acetoacetate, Isoamyl Alcohol, Isoamyl Benzoate, Isoamyl Butyrate, Isoamyl Cinnamate, Isoamyl Formate, Isoamyl Hexanoate, Isoamyl Isobutyrate, Isoamyl Isothiocyanate, Isoamyl Isovalerate, Isoamyl Laurate, Isoamyl Levulinate, Isoamyl Nonanoate, Isoamyl Octanoate, Isoamyl Phenethyl Ether, Isoamyl Phenylacetate, Isoamyl Propionate, Isoamyl Pyruvate, Isoamyl Salicylate, Isoamyl-2-Methylbutyrate, Isoascorbic Acid, Isoborneol, Isobornyl 2-Methylbutyrate, Isobornyl Acetate, Isobornyl Formate, Isobornyl Isobutyrate, Isobornyl Isovalerate, Isobornyl Propionate, Isobutanal Propyleneglycol Acetal, Isobutyl 10-Undecenoate, Isobutyl 2-Butenoate, Isobutyl 2-Furanpropionate, Isobutyl 3-(2-Furan)Propionate, Isobutyl Acetate, Isobutyl Acetoacetate, Isobutyl Alcohol, Isobutyl Angelate, Isobutyl Anthranilate, Isobutyl Benzoate, Isobutyl Benzyl Carbinol, Isobutyl Butyrate, Isobutyl Cinnamate, Isobutyl Formate, Isobutyl Heptanoate, Isobutyl Hexanoate, Isobutyl Isobutyrate, Isobutyl Isothiocyanate, Isobutyl N-Methylanthranilate, Isobutyl Phenylacetate, Isobutyl Propionate, Isobutyl Salicylate, Isobutylamine, Isobutylthiazole, Isobutyraldehyde, Isobutyric Acid, Isocineole, Isodecylaldehyde, Isoeugenol, Isoeugenyl Acetate, Isoeugenyl Benzyl Ether, Isoeugenyl Ethyl Ether, Isoeugenyl Formate, Isoeugenyl Methyl Ether, Isoeugenyl Phenylacetate, Isojasmone, Isomalt, Isopentyl Alcohol, Isopentyl-2-Methylbutyrate, Isopentylamine, Isopentylidene Isopentylamine, Isophorone, Isoprenyl Acetate, Isopropanol, Isopropenyl Acetate, Isopropenylpyrazine, Isopropyl 2-Methylbutyrate, Isopropyl Acetate, Isopropyl Alcohol, Isopropyl Benzoate, Isopropyl Butyrate, Isopropyl Cinnamate, Isopropyl Formate, Isopropyl Hexanoate, Isopropyl Isobutyrate, Isopropyl Isothiocyanate, Isopropyl Isovalerate, Isopropyl Myristate, Isopropyl Phenylacetate, Isopropyl Propionate, Isopropyl Tiglate, Isopropylamine, Isopulegol, Isopulegone, Isopulegyl Acetate, Isoquinoline, Isovaleraldehyde, Isovaleric Acid, Japanese Camphor White Oil, Jasmalone, Karaya Gum, L-8-P-Menthene-1-2-Epoxide, Lactic Acid, Lactose, L-Arabinose, L-Arginine, L-Asparagine, L-Aspartic Acid, Lauric Acid, Lauric Aldehyde, Lauryl Acetate, Lauryl Alcohol, L-Bornyl Acetate, L-Cysteine, L-Cystine, Leaf Alcohol, Leaf Aldehyde, Lepidine, Levulinic Acid, Levulose, L-Glutamic Acid, L-Glutamine, L-Histidine, Licorice, Linalool, Linalyl Acetate, Linalyl Anthranilate, Linalyl Benzoate, Linalyl Butyrate, Linalyl Cinnamate, Linalyl Formate, Linalyl Hexanoate, Linalyl Isobutyrate, Linalyl Isovalerate, Linalyl Octanoate, Linalyl Phenylacetate, Linalyl Propionate, Linoleic And Linolenic Acid, Lipase, L-Isoleucine, L-Leucine, L-Lysine, L-Malic Acid, L-Menthol Ethylene Glycol Carbonate, L-Menthyl (R S)-3-Hydroxybutyrate, L-Menthyl Acetoacetate, L-Menthyl Butyrate, L-Menthyl L-Lactate, L-Menthyl Methyl Ether, L-Methionine, L-Methionylglycine, L-Monomenthyl Glutarate, Locust Bean Gum, L-Ornithine Monochlorohydrate, L-Phenylalanine, L-Piperitone, L-Proline, L-Serine, L-Threonine, L-Tyrosine, Lysin Hydrochloride, Magnesium Carbonate, Magnesium Di-L-Glutamate, Magnesium Gluconate, Magnesium Glutamate, Magnesium Oxide, Magnesium Sulfate, Magnesium Sulphate, Magnolol, Malic Acid, Malt Extract, Malt Syrup, Maltol, Maltyl Isobutyrate, Mannitol, M-Cresol, M-Dimethoxybenzene, Melilotal, Menthadienol, Menthadienyl Acetate, Menthofuran, Menthol, Menthol-Propylene Glycol Carbonate, Menthone, Menthone-8-Thioacetate, Menthyl Acetate, Menthyl Formate, Menthyl Isovalerate, Menthyl Propionate, Menthyl Pyrrolidone Carboxylate, Menthyl Valerate, Methanethiol, Methional, Methionyl Butyrate, Methoxybenzene, Methoxypyrazine, Methoxystyryl Isopropyl Ketone, Methyl (Methylthio)Acetate, Methyl 10-Undecenoate, Methyl 1-Acetoxycyclohexyl Ketone, Methyl 1-Propenyl Disulfide, Methyl 1-Propenyl Sulfide, Methyl -2-4-Decadienoate, Methyl 2-Furoate, Methyl 2-Hexanoate, Methyl 2-Hydroxy-4-Methylpentanoate, Methyl 2-Methyl-2-Propenoate, Methyl 2-Methyl-3-Furyl Disulfide, Methyl 2-Methylbutyrate, Methyl 2-Methylpentanoate, Methyl 2-Methylphenyl Disulfide, Methyl 2-Nonenoate, Methyl 2-Nonynoate, Methyl 2-Octynoate, Methyl 2-Oxo-3-Methylpentanoate, Methyl 2-Pyrrolyl Ketone, Methyl 2-Undecynoate, Methyl 3-(Furfurylthio)Propionate, Methyl 3-(Methylthio)Butanoate, Methyl 3-7-Dimethyl-6-Octenoate, Methyl 3-7-Dimethyl-6-Octenoate, Methyl 3-Acetoxy-2-Methylbutyrate, Methyl 3-Acetoxyoctanoate, Methyl 3-Hexenoate, Methyl 3-Hydroxybutyrate, Methyl 3-Hydroxyhexanoate, Methyl 3-Mercaptobutanoate, Methyl 3-Methyl-1-Butenyl Disulfide, Methyl 3-Methylthiopropionate, Methyl 3-Nonenoate, Methyl 3-Phenylpropionate, Methyl 4-(Methylthio)Butyrate, Methyl 4-Methylvalerate, Methyl 4-Pentenoate, Methyl 4-Phenylbutyrate, Methyl 5-Acetoxyhexanoate, Methyl 5-Octenoate, Methyl 9-Undecenoate, Methyl Acetate, Methyl Amyl Ketone, Methyl Anisate, Methyl Anthranilate, Methyl Benzoate, Methyl Benzyl Disulfide, Methyl Beta-Naphthyl Ketone, Methyl Beta-Phenylglycidate, Methyl Butyrate, Methyl Cellulose, Methyl Cinnamate, Methyl Cis-4-Octenoate, Methyl Cyclohexadiene And Methylene Cyclohexene, Methyl Cyclohexanecarboxylate, Methyl Decyne Carbonate, Methyl Dihydroabietate, Methyl Dihydrojasmonate, Methyl Disulfide, Methyl Ethyl Acetaldehyde, Methyl Ethyl Carbinol, Methyl Ethyl Ketone, Methyl Ethyl Sulfide, Methyl Ethyl Trisulfide, Methyl Eugenol, Methyl Furfuryl Disulfide, Methyl Heptanoate, Methyl Heptine Carbonate, Methyl Heptyl Ketone, Methyl Hexanoate, Methyl Hexyl Acetaldehyde, Methyl Hexyl Ether, Methyl Hexyl Ketone, Methyl Hydrocinnamate, Methyl Isobutanethioate, Methyl Isobutyl Ketone, Methyl Isobutyrate, Methyl Isoeugenol, Methyl Isopentyl Disulfide, Methyl Isothiocyanate, Methyl Isovalerate, Methyl Jasmonate, Methyl Laurate, Methyl Levulinate, Methyl Linoleate (48%) Methyl Linolenate (52%) , Methyl Linoleate And Methyl Linolenate Mixture, Methyl Mercaptan, Methyl Myristate, Methyl N-Acetylanthranilate, Methyl N-Formylanthranilate, Methyl Nicotinate, Methyl N-Methylanthranilate, Methyl N-N-Dimethylanthranilate, Methyl Nonanoate, Methyl Nonyl Acetaldehyde, Methyl Nonyl Ketone, Methyl Octanoate, Methyl Octyl Sulfide, Methyl O-Methoxybenzoate, Methyl O-Methoxybenzoate, Methyl Para-Hydroxybenzoate, Methyl Pentyl Ketone, Methyl Phenethyl Ether, Methyl Phenyl Disulfide, Methyl Phenyl Ketone, Methyl Phenyl Sulfide, Methyl Phenylacetate, Methyl P-Hydroxybenzoate, Methyl Propionate, Methyl Propyl Disulfide, Methyl Propyl Ketone, Methyl Propyl Trisulfide, Methyl P-Tert-, Methyl P-Tolyl Ketone, Methyl Salicylate, Methyl Sorbate, Methyl Styryl Carbinol, Methyl Sulfide, Methyl Thiobutyrate, Methyl Trans-2-Octenoate, Methyl Valerate, Methyl-2-Butenal, Methyl-3-Methylthiopropionate, Methyl-3-Pentenoic Acid, Methyl-5-Hepten-2-Ol, Methylallyl Butyrate, Methylbenzyl Acetate, Methylbutyric Acid, Methylcellulose, Methylcyclopentenolone, Methylcyclo-Pentenolone, Methylene Chloride, Methylethylacetaldehyde, Methylheptyl Ketone, Methylhexylacetaldehyde, Methyloctyne Carbonate, Methylparaben, Methylsulfinylmethane, Methylthio 2-(Acetyloxy)Propionate, Methylthio 2-(Propionyloxy)Propionate, Methylthiomethyl Butyrate, Methylthiomethyl Hexanoate, Methylthiomethylmercaptan, Methylvaleric Acid, Microcrystalline Cellulose, Milled Rice, Mintlactone, Modified Food Starch, Modified Pectin, Mono And Diglycerides Of Fatty Acids, Mono-Menthyl Succinate, Monosodium Succinate, Msg, Mustard Oil, Myrcene, Myrcenyl Methyl Ether, Myricitrin, Myristaldehyde, Myristic Acid, Myristicin, Myrtenol, Myrtenyl Acetate, N- Hexyl Phenylacetate, N-(1-1-Dimethyl-2-Hydroxyethyl)-2-2-Diethylbutanamide, N-(2-(Pyridin-2-Yl)Ethyl)-3-P-Menthanecarboxamide, N-(2-Hydroxyethyl)-2-3-Dimethyl 2-Isopropylbutanamide, N-(2-Methylcyclohexyl)-2-3-4-5-6-Pentafluorobenzamide, N1-(2-3-Dimethoxybenzyl)-N2-(2-(Pyridin-2-Yl)Ethyl)Oxalamide, N1-(2-4-Dimethoxybenzyl)-N2-(2-(Pyridin-2-Yl)Ethyl)Oxalamide, N1-(2-Methoxy-4-Methylbenzyl)-N2-(2-(5-Methylpyridin-2-Yl)Ethyl)Oxalamide, N1-(2-Methoxy-4-Methylbenzyl)-N2-(2-(Pyridin-2-Yl)Ethyl)Oxalamide, N-3-7-Dimethyl-2-6-Octadienyl Cyclopropylcarboxamide, N-Acetyl Glutamate, N-Amyl Butyrate, N-Amyl Formate, N-Amyl Heptanoate, N-Amyl Hexanoate, N-Amyl Octanoate, Naringin Dihydrochalcone, Naringin Extract, N-Benzoylanthranilic Acid, N-Butyl 2-Methylbuytyrate, N-Cyclopropyl-2-6-Nonadienamide, N-Cyclopropyl-5-Methyl-2-Isopropylcyclohexanecarbonecarboxamide, Neomenthol, Neotame, Nerol, Neryl Acetate, Neryl Butyrate, Neryl Formate, Neryl Isobutyrate, Neryl Isovalerate, Neryl Propionate, N-Ethyl 2-Isopropyl-5-Methylcyclohexanecarboxamide, N-Ethyl-2-2-Diisopropylbutanamide, N-Ethyl-2-6-Nonadienamide, N-Ethyl-2-Isopropyl-5-Methylcyclohexane Carboxamide, N-Ethyl-5-Methyl-2-(Methylethenyl)Cyclohexanecarboxamide, N-Furfurylpyrrole, N-Gluconyl Ethanolamine, Niacinamide, N-Isobutyl (E-E)-2-4-Decadienamide, Nitrous Oxide, N-Lactoyl Ethanolamine, N-N-Dimethyl Menthyl Succinamide, N-N-Dimethylphenethylamine, Nona-2-4-6-Trienal, Nona-2-Trans-6-Cis-Dienal, Nonanal, Nonane Diacetate, Nonanoic Acid, Nonanoyl 4-Hydroxy-3-Methoxybenzylamide, Nonyl Acetate, Nonyl Alcohol, Nonyl Isovalerate, Nonyl Octanoate, Nootkatone, N-P-Benzeneacetonitrile Menthanecarboxamide, O-(Ethoxymethyl)Phenol, O-(Methylthio)Phenol, O-Anisaldehyde, Ocimene, O-Cresol, O-Cresyl Acetate, Octadecalactone, Octahydrocoumarin, Octalactone, Octanal, Octanoic Acid, Octyl 2-Furoate, Octyl 2-Methylbutyrate, Octyl Acetate, Octyl Alcohol, Octyl Butyrate, Octyl Formate, Octyl Heptanoate, Octyl Isobutyrate, Octyl Octanoate, Octyl Phenylacetate, Octyl Propionate, Odium Sulfate, O-Ethyl S-(2-Furylmethyl)Thiocarbonate, Oleic Acid, Omega-6-Hexadecenolactone, Omega-Pentadecalactone, O-Methoxybenzaldehyde, O-Methoxycinnamaldehyde, O-Methylanisole, O-Propylphenol, Orin Lactone, O-Toluenethiol, O-Tolyl Acetate, O-Tolyl Isobutyrate, O-Tolyl Salicylate, Palmitic Acid, P-Alpha-Alpha-Trimethylbenzyl Alcohol, P-Alpha-Dimethylbenzyl Alcohol, P-Alpha-Dimethylstyrene, P-Anisaldehyde, Papain, Paraffin Wax, Paraldehyde, Partially Hydrogenated Methyl Ester Of Rosin, Partially Hydrogenated Methyl Ester Rosin, P-Cresol, P-Cresyl Acetate, P-Cymene, P-Dimethoxybenzene, Peach Aldehyde, Peanut Oil, Pectin, Pectinase From Aspergillus Niger, Pectinase From Bacillus Subtilis, Pectins, Pelargonic Acid, Pelargonic Aldehyde, Pelargonyl Vanillylamide, Pentadecanoic Acid, Pentadecanolide, Pentanal, Pentanoic Acid, Pentyl 2-Furyl Ketone, Pentyl Alcohol, Pentylamine, Peptone, Perillaldehyde, Perillyl Acetate, Perillyl Alcohol, P-Ethoxybenzaldehyde, P-Ethylphenol, Phenethyl 2-Furoate, Phenethyl 2-Methylbutyrate, Phenethyl Acetate, Phenethyl Alcohol, Phenethyl Anthranilate, Phenethyl Benzoate, Phenethyl Butyrate, Phenethyl Cinnamate, Phenethyl Formate, Phenethyl Hexanoate, Phenethyl Isobutyrate, Phenethyl Isothiocyanate, Phenethyl Isovalerate, Phenethyl Mercaptan, Phenethyl Octanoate, Phenethyl Phenylacetate, Phenethyl Propionate, Phenethyl Salicylate, Phenethyl Senecioate, Phenethyl Tiglate, Phenethylamine, Phenol, Phenoxyacetic Acid, Phenoxyethyl Isobutyrate, Phenyl Acetate, Phenyl Butyrate, Phenyl Disulfide, Phenyl Salicylate, Phenylacetaldehyde, Phenylacetic Acid, Phenyldimethylcarbinyl Isobutyrate, Phenylethyl Anthranilate, Phenylethyl Carbinol, Phenylethyl Methyl Carbinol, Phenylethyl Methyl Carbinyl Acetate, Phenylethyl Methyl Ethyl Carbinol, Phenylpropyl Propionate, Phosphates, Phosphoric Acid, P-Hydroxybenzyl Acetone, Pine Tar Oil, Pinocarveol, Pinocarvyl Isobutyrate, Piperazine, Piperidine, Piperine, Piperitenone, Piperitone, Piperitone, Piperonal, Piperonyl Acetate, Piperonyl Isobutyrate, P-Isopropyl Benzaldehyde, P-Isopropylacetophenone, P-Isopropylbenzyl Alcohol, P-Isopropylphenylacetaldehyde, P-Menth-1-4(8)-Dien-3-One, P-Menth-1-En-3-Ol, P-Menth-1-En-9-Al, P-Menth-1-En-9-Ol, P-Menth-3-En-1-Ol, P-Menth-8-En-1-Ol, P-Mentha-1-3-Diene, P-Mentha-1-4-Diene, P-Mentha-1-8-Dien-7-Al, P-Mentha-1-8-Dien-7-Ol, P-Mentha-1-8-Dien-7-Yl Acetate, P-Mentha-6-8-Dien-2-Ol, P-Mentha-8-Thiol-3-One, P-Menthan-2-Ol, P-Menthan-2-One, P-Menthan-7-Ol, P-Menthane-3-8-Diol, P-Methoxy-Alpha-Methyl-Cinnamaldehyde, P-Methoxybenzaldehyde, P-Methoxybenzyl Alcohol, P-Methoxycinnamaldehyde, P-Methoxytoluene, P-Methylacetophenone, P-Methylanisole, P-Methylbenzylacetone, P-Methylcinnamaldehyde, P-Methylhydratropic Aldehyde, Polylimonene, Polyoxyethylene (20) Sorbitan Monolaurate, Polyoxyethylene (20) Sorbitan Monooleate, Polyoxyethylene (20) Sorbitan Monostereate  Polysorbate, Potassium 2-(1'-Ethoxy)Ethoxypropanoate, Potassium 5'-Inosinate, Potassium Acetate, Potassium Alginate, Potassium Chloride, Potassium Citrate, Potassium Glutamate, Potassium Inosinate, Potassium Lactate, Potassium Nitrate, Potassium Sorbate, Potassium Sulfate, Potassium Sulphate, Potato Starch, P-Propyl Anisole, P-Propylanisole, P-Propylphenol, Prenyl Acetate, Prenyl Benzoate, Prenyl Caproate, Prenyl Formate, Prenyl Isobutyrate, Prenyl Thioacetate, Prenylthiol, Propanethiol, Propenyl Propyl Disulfide, Propenylguaethol, Propionaldehyde, Propionic Acid, Propiophenone, Propyl 2-4-Decadienoate, Propyl 2-Furanacrylate, Propyl 2-Furoate, Propyl 2-Mercaptopropionate, Propyl 2-Methyl-3-Furyl Disulfide, Propyl Acetate, Propyl Alcohol, Propyl Benzoate, Propyl Butyrate, Propyl Cinnamate, Propyl Disulfide, Propyl Formate, Propyl Furfuryl Disulfide, Propyl Heptanoate, Propyl Hexanoate, Propyl Isobutyrate, Propyl Isovalerate, Propyl Levulinate, Propyl Mercaptan, Propyl Phenylacetate, Propyl Propane Thiosulfonate, Propyl Propionate, Propyl Pyruvate, Propyl Sorbate, Propyl Thioacetate, Propylamine, Propylene Glycol, Propyleneglycol, Propylidenephthalide, Propylparaben, Protease, Protein Hydrolysate, P-Tolyl 3-Methylbutyrate, P-Tolyl Acetate, P-Tolyl Isobutyrate, P-Tolyl Laurate, P-Tolyl Octanoate, P-Tolyl Phenylacetate, P-Tolylacetaldehyde, Pulegone, P-Vinylguaiacol, P-Vinylphenol, Pyrazine, Pyrazinyl Methyl Sulfide, Pyridine, Pyroligneous Acid Extract, Pyrrole, Pyrrolidine, Pyruvaldehyde, Pyruvic Acid, Quillaia, Red Algae, Rennet, Resorcinol, Rhodinal, Rhodinol, Rhodinyl Acetate, Rhodinyl Butyrate, Rhodinyl Formate, Rhodinyl Isobutyrate, Rhodinyl Isovalerate, Rhodinyl Phenylacetate, Rhodinyl Propionate, Riboflavin, Ricinoleic Acid Linoleic Acid And Oleic Acid, Rum Ether, Saffron Extract, Safrole, Salicylaldehyde, S-Allyl-L-Cysteine, Santalol, Santalol, Santalyl Acetate, Santalyl Phenylacetate, Sclareolide, Sec-Butyl Ethyl Ether, Sec-Butyl Ethyl Ether, Sec-Butylamine, S-Ethyl 2-Acetylamino Ethanethioate, S-Furfuryl Thioacetate, S-Furfuryl Thioformate, S-Furfuryl Thiopropionate, S-Isopropyl 3-Methylbut-2-Enethioate, Skatole, S-Methyl 2-Methylbutanethioate, S-Methyl 4-Methylpentanethioate, S-Methyl Benzothioate, S-Methyl Hexanethioate, S-Methyl Propanethioate, S-Methyl Thioacetate, S-Methyl Thiofuroate, Sodium 2-(4-Methoxyphenoxy)Propanoate, Sodium 3-Mercapto-Oxopropionate, Sodium 3-Methoxy-4-Hydroxycinnamate, Sodium 4-(Methylthio)-2-Oxobutanoate, Sodium 4-Methoxybenzoyloxyacetate, Sodium Alginate, Sodium And Potassium Nitrates, Sodium And Potassium Nitrites, Sodium Benzoate, Sodium Bicarbonate, Sodium Calcium Alginate, Sodium Gluconate, Sodium Glutamate, Sodium Hydroxide, Sodium Lactate, Sodium L-Aspartate, Sodium Metaphosphate, Sodium Nitrate, Sodium Nitrite, Sodium Phosphate Dibasic, Sodium Phosphate Monobasic, Sodium Salt Of Carrageenan, Sodium Salt Saccharin, Sodium Succinate, Sodium Sulphate, Sopropylideneglyceryl 5-Hydroxydecanoate And Delta-Decalactone, Sorbitan Monostearate, Spiro(2-4-Dithia-1-Methyl-8-Oxabicyclo(3.3.0)Octane-3-3'-(1'-Oxa-2'-Methyl)Cyclopentane), Stearic Acid, Styralyl Acetate, Styralyl Alcohol, Styralyl Butyrate, Styralyl Formate, Styralyl Isobutyrate, Styralyl Propionate, Succinic Acid, Sucrolase, Sucrose Acetate Isobutyrate, Sucrose Octaacetate, Sulcatone, Sulfonated Anthracite Coal, Sulfur Dioxide, Sulfuric Acid, Sulphur Dioxide, Sulphuric Acid, Sweet Blackberry Leaves Extract, Tannic Acid, Tannins, Tartaric Acid, Taurine, Terpinolene, Terpinyl Acetate, Terpinyl Anthranilate, Terpinyl Butyrate, Terpinyl Cinnamate, Terpinyl Formate, Terpinyl Isobutyrate, Terpinyl Isovalerate, Terpinyl Propionate, Tetradecalactone, Tetradecanal, Tetrahydro-4-Methyl-2-(2-Methylpropen-1-Yl)Pyran, Tetrahydrofurfuryl Acetate, Tetrahydrofurfuryl Alcohol, Tetrahydrofurfuryl Butyrate, Tetrahydrofurfuryl Cinnamate, Tetrahydrofurfuryl Propionate, Tetrahydrogeraniol, Tetrahydrolinalool, Tetramethyl Ethylcyclohexenone, Thaumatin, Theaspirane, Thiamine, Thiazole, Thioacetic Acid, Thiogeraniol, Thiophenethiol, Thiophenol, Thujyl Alcohol, Thymol, Tocopherols, Tolualdehyde Glyceryl Acetal, Tolualdehydes, Tonkalide, Tragacanth Gum, Trans-2-Butenoic Acid Ethylester, Trans-2-Heptenal, Trans-2-Hexenal Glyceryl Acetal, Trans-2-Hexenal Propylene Glycol Acetal, Trans-2-Hexenoic Acid, Trans-2-Hexenyl 2-Methylbutyrate, Trans-2-Hexenyl Butyrate, Trans-2-Hexenyl Isovalerate, Trans-2-Hexenyl Pentanoate, Trans-2-Hexenyl Propionate, Trans-2-Methyl-2-Butenoic Acid, Trans-2-Nonen-1-Ol, Trans-2-Nonenyl Acetate, Trans-2-Octen-1-Yl Acetate, Trans-2-Octen-1-Yl Butanoate, Trans-2-Tridecenol, Trans-3-Heptenyl 2-Methyl Propanoate, Trans-3-Heptenyl Acetate, Trans-3-Hexenol, Trans-3-Hexenyl Acetate, Trans-3-Nonen-1-Ol, Trans-4-Hexenal, Trans-4-Octenoic Acid, Trans-4-Tert-Butylcyclohexanol, Trans-5-Dodecenal, Trans-5-Octenal, Trans-6-Octenal, Trans-Anethole, Trans-Tetradec-4-Enal, Trans-Trans-2-4-Dodecadienal, Trans-Trans-2-4-Octadienal, Triacetin, Tributyl Acetylcitrate, Tributyrin, Trichlorogalactosucrose, Tridec-5-Enal, Tridecanal, Tridecanedioic Acid Cyclic Ethylene Glycol Diester, Tridecanoic Acid, Triethyl Citrate, Triethylamine, Triethylthialdine, Trilobatin, Trimethylamine, Trimethyloxazole, Tripropylamine, Trithioacetone, Tuberose Lactone, Tween 20, Undecanal, Undecanoic Acid, Undecen-1-Ol, Undecenal, Undecenoic Aldehyde, Undecenyl Acetate, Undecyl Alcohol, Undecylenic Alcohol, Undecylenic Aldehyde, Valencene, Valeraldehyde, Valeric Acid, Vanillin, Vanillyl Alcohol, Vanillyl Butyl Ether, Vanillyl Ethyl Ether, Vanillylidene Acetone, Veratraldehyde, Verbenol, Verbenone, Vetiverol, Vetiveryl Acetate, Wheat Gluten, Wheat Starch, Whey, Wild Cherry Bark Extract, Yeast Autolysate, Yeast Extract, Yeasts, Yuzunone, Zinc Acetate, Zingerone |
| Preservatives | 200, 201, 202, 203, 209, 210, 211, 212, 213, 214, 215, 216, 217, 218, 219, 220, 221, 222, 223, 224, 225, 226, 227, 228, 230, 231, 232, 233, 234, 235, 236, 237, 238, 239, 240, 241, 242, 243, 249, 250, 251, 252, 260, 261, 262, 265, 266, 280, 281, 282, 283, 290, 300, 301, 302, 303, 304, 305, 307, 308, 309, 310, 311, 312, 313, 314, 315, 316, 317, 318, 319, 320, 321, 322, 323, 324, 325, 326, 330, 334, 335, 336, 337, 338, 384, 385, 386, 387, 388, 389, 390, 392, Acetic Acid, Acrolein, Ammonium Chloride, Ammonium Persulfate, Anoxomer, Antimicrobial, Antioxidant, Ascorbic Acid, Ascorbyl Palmitate, Ascorbyl Stearate, Benzoic Acid, Bha, Bht, Bisabolene, Butylated Hydroxyanisole, Butylated Hydroxytoluene, Calcium Ascorbate, Calcium Benzoate, Calcium Disodium Ethylenediaminetetraacetate, Calcium Formate, Calcium Hydrogen Sulfite, Calcium Isoascorbate, Calcium Propionate, Calcium Sorbate, Calcium Sulfite, Carbon Dioxide, Catalase From Aspergillus Niger, Catalase From Bovine Liver, Catalase From Penicillium Notatum, Chlorine, Citric Acid, Dehydroacetic Acid, Dibromo-3-Nitrilopropionamide, Dilauryl Thiodipropionate, Dimethyl Dicarbonate, Diphenyl, Dipotassium Tartrate, Disodium Cyanodithioimidocarbonate, Disodium Ethylenebisdithiocarbamate, Disodium Ethylenediaminetetraacetate, Distearyl Thiodipropionate, Dodecyl Gallate, Egg White Lysozyme, Erythorbic Acid, Ethoxyquin, Ethyl Alcohol, Ethyl Gallate, Ethyl Para-Hydroxybenzoate, Ethylene Oxide-Nlfg, Formaldehyde, Formic Acid, Fumaric Acid, Guaiac Resin, Gum Guaicum, Heptyl Para-Hydroxybenzoate, Hexamethylene Tetramine, Hydrogen Peroxide, Isoascorbate, Isopropyl Citrates, Lactic Acid, Lauric Arginate Ethyl Ester, Lecithin, Listeria-Specific Bacteriophage Preparation, L-Malic Acid, Methyl Benzoate, Methyl Esters Of Fatty Acids, Methyl Para-Hydroxybenzoate, Methyl P-Hydroxybenzoate, Monopotassium Tartrate, Monosodium Tartrate, Natamycin, Nisin, Nitrate, Nitrite, Octyl Gallate, O-Phenylphenol, Ortho-Phenylphenol, Oxystearin, Ozone, Phosphoric Acid, Potassium Acetate, Potassium Ascorbate, Potassium Benzoate, Potassium Bisulfite, Potassium Diacetate, Potassium Isoascorbate, Potassium Lactate, Potassium Metabisulfite, Potassium Nitrate, Potassium N-Methyldithiocarbamate, Potassium Oleate, Potassium Propionate, Potassium Sodium L(+)-Tartrate, Potassium Sorbate, Potassium Stearate, Potassium Sulfite, Propionic Acid, Propyl Gallate, Propyl Para-Hydroxybenzoate, Propyl P-Hydroxybenzoate, Propylene Glycol, Rosemary Extract, Saccharin, Sodium Acetate, Sodium Ascorbate, Sodium Benzoate, Sodium Bisulfite, Sodium Chloride, Sodium Chlorite, Sodium Dehydroacetate, Sodium Diacetate, Sodium Dimethyldithiocarbamate, Sodium Erythorbate, Sodium Ethyl Para-Hydroxybenzoate, Sodium Formate, Sodium Hydrogen Sulfite, Sodium Lactate, Sodium Metabisulfite, Sodium Methyl Para-Hydroxybenzoate, Sodium Ortho-Phenylphenol, Sodium Phosphate, Sodium Propionate, Sodium Propyl Para-Hydroxybenzoate, Sodium Sorbate, Sodium Sulfite, Sorbic Acid, Sulfur Dioxide, Tartaric Acid, Tartrate, Tertiary Butylhydroquinone, Thiabendazole, Thiodipropionic Acid, Tocopherol |
| Non-nutritive sweeteners | 420, 421, 950, 951, 952, 953, 954i, 955, 956, 957, 960, 961, 962, 964, 965i, 966, 967, 968, 969, Acesulfame, Advantame, Alitame, Altern, Aspartame, Brazzein, Candy Leaf, Candyleaf, Curculin, Cweet, Cyclamate, Cyclamic Acid, Enliten, Equal, Erythritol, Galactitol, Glucitol, Hydrogenated Isomaltulose, Insta Sweet, Instasweet, Isomalt, Kaltame, Lactitol, Lumbah, Luo Han , Luohan, Mabinlin, Maltitol, Mannitol, Monatin, Monellin, Monk Fruit, Natra Taste, Natrataste, Necta Sweet, Nectasweet, Neohesperidine Dihydrochalcone, Neotame, Nutra Sweet, Nutrasweet, Osladin, Oubli, Pentadin, Polyglycitol, Purevia, Reb A, Reb-A, Rebaudioside A, Rebiana, Saccharin, Sorbitol, Splenda, Stevia, Steviol, Stevioside, Sucralose, Sucrolase, Sugar Leaf, Sugar Twin, Sugarleaf, Sugartwin, Sunett, Sweet N Low, Sweet Nlow, Sweet One, Sweet’n Low, Sweetleaf, Sweetn Low, Sweetnlow, Sweetone, Syclamate, Thaumatin, Trichlorogalactosucrose, Truvia, Twin Sweet, Twinsweet, Xylitol |
| Other Additives | 1001, 1101, 1103, 1200, 1201, 1203, 1204, 1205, 1209, 1400, 1401, 1402, 1403, 1404, 1405, 1410, 1411, 1412, 1413, 1414, 1420, 1422, 1423, 1440, 1442, 1443, 1450, 1451, 1452, 1503, 1505, 1518, 1520, 1521, 181, 263, 290, 322, 325, 326, 327, 331, 332, 333, 335, 337, 339, 340, 341i, 342i, 343, 353, 383, 400, 401, 402, 403, 404, 405, 406, 407, 408, 409, 410, 411, 412, 413, 414, 415, 416, 417, 418, 419, 420, 421, 422, 423, 424, 425, 426, 427, 428, 429, 430, 431, 432, 433, 434, 435, 436, 437, 440, 441, 442, 443, 444, 445, 446, 450, 451, 452, 458, 460i, 461, 462, 463, 464, 465, 466, 467, 468, 470i, 471, 472, 473, 474, 475, 476, 477, 478, 479, 480, 481, 482, 484, 485, 486, 487, 488, 489, 491, 492, 493, 494, 495, 500i, 508, 509, 516, 518, 520, 525, 539, 541, 542, 551, 553iii, 558, 576, 578, 621, 624, 631, 900a, 900b, 901, 902, 903, 904, 905b, 905c, 905d, 905e, 906, 907, 909, 910, 911, 915, 941, 942, 953, 965i, 966, 967, 999, Acacia Gum, Acetic And Fatty Acid Esters Of Glycerol, Acetylated Distarch Adipate, Acetylated Distarch Glycerol, Acetylated Distarch Phosphate, Acetylated Monoglycerides, Acetylated Oxidized Starch, Acid Treated Starch, Acid-Treated Starch, Agar, Albumin, Alginic Acid, Alkaline Treated Starch, Alkaline-Treated Starch, Aluminium Sulfate, Aluminium Sulphate, Aluminum Caprate, Aluminum Caprylate, Aluminum Laurate, Aluminum Myristate, Aluminum Oleate, Aluminum Palmitate, Aluminum Salts Of Fatty Acids, Aluminum Stearate, Aluminum Sulfate, Aluminum Sulphate, Amidated Pectin, Ammonium Alginate, Ammonium Dihydrogen Phosphate, Ammonium Glutamate, Ammonium Polyphosphate, Ammonium Salt Of Carrageenan, Ammonium Salt Of Furcelleran, Ammonium Salts Of Phosphatidic Acid, Antifoam, Arabic Gum, Arabinogalactan, Arrowroot Starch, Ascorbic Acid, Bakers Yeast Glycan, Basic Methacrylate Copolymer, Beeswax, Bentonite, Benzoin Gum, Benzoyl Peroxide Modified Lecithin, Beta-1-3-Glucan, Bleached Starch, Bmc, Bone Phosphate, Brominated Vegetable Oil, Brown Mustard Extract, Bulking Agent, Calcium Acetate, Calcium Alginate, Calcium Caprate, Calcium Caprylate, Calcium Caseinate, Calcium Chloride, Calcium Dihydrogen Diphosphate, Calcium Dihydrogen Phosphate, Calcium Gluconate, Calcium Glycerophosphate, Calcium Hydrogen Phosphate, Calcium Lactate, Calcium Lactylate, Calcium Laurate, Calcium Myristate, Calcium Oleate, Calcium Oleyl Lactylate, Calcium Palmitate, Calcium Phosphate Dibasic, Calcium Phosphate Monobasic, Calcium Phosphate Tribasic, Calcium Polyphosphate, Calcium Salt Of Carrageenan, Calcium Salt Of Furcelleran, Calcium Salts Of Fatty Acids, Calcium Stearate, Calcium Stearoyl Fumarate, Calcium Stearoyl Lactylate, Calcium Stearoyl-2 Lactylate, Calcium Sulfate, Calcium Sulphate, Candelilla Wax, Carbon Dioxide, Carboxymethyl Cellulose, Carboxymethyl Hydroxyethyl Cellulose, Carnauba Wax, Carob Bean Gum, Carrageenan, Casein, Cassia Gum, Castor Oil, Cellulose, Choline Acetate, Choline Carbonate, Choline Chloride, Choline Citrate, Choline Lactate, Choline Salts And Esters, Choline Tartrate, Chymosin Preparation, Citric And Fatty Acid Esters Of Glycerol, Corn Starch, Cornstarch, Croscaramellose, Curdlan, Deoxycholic Acid, Desoxycholic, Dextrans, Dextrin, Dextrose, Diacetyltartaric And Fatty Acid Esters Of Glycerol, Diammonium Hydrogen Phosphate, Diammonium Phosphate, Dicalcium Diphosphate, Dihydroxycholanoic Acid, Dimagnesium Phosphate, Dioctyl Sodium Sulfosuccinate, Dioctyl Sodium Sulphosuccinate, Dipotassium Diphosphate, Dipotassium Hydrogen Phosphate, Dipotassium Phosphate, Dipotassium Pyrophosphate, Disodium 5'-Inosinate, Disodium Citrate, Disodium Diphosphate, Disodium Hydrogen Phosphate, Disodium Inosinate, Disodium Iron Edta, Disodium Monohydrogen Citrate, Disodium Phosphate, Distarch Glycerol, Distarch Oxypropanol, Distarch Phosphate, Emulsifier, Emulsifying Salt, Enzyme Activated Soy Protein Concentrate, Enzyme Modified Lecithin, Enzyme Treated Starch, Enzyme-Modified Lecithin, Enzyme-Modified Whole Milk Powder, Epoxidized Soybean Oil, Ethoxylated Mono- And Di- Glycerides, Ethoxylated Mono And Diglycerides, Ethyl Alcohol, Ethyl Cellulose, Ethyl Hydroxyethyl Cellulose, Ethylcellulose, Ethylene Oxide, Ferrous Sulfate, Furcelleran, Gamma Cyclodextrin, Gamma-Cyclodextrin, Gelatin, Gelatine, Gellan Gum, Gelling Agent, Ghatti Gum, Glazing Agent, Glycerin, Glycerol, Glycerol Erithrytol Ester Of Colophane, Glycerol Erithrytol Esters Of Colophane, Glycerol Ester Of Gum Rosin, Glycerol Ester Of Rosin, Glycerol Ester Of Tall Oil Rosin, Glycerol Ester Of Wood Rosin, Glycerol Esters Of Gum Rosin, Glycerol Esters Of Rosin, Glycerol Esters Of Tall Oil Rosin, Glycerol Esters Of Wood Resin, Glycerol Esters Of Wood Rosin, Glycerol Monostearate, Glyceryl Lactooleate, Glyceryl Lactopalmitate, Glyceryl Monooleate, Glyceryl Monostearate, Guar, Gum Arabic, Gum Ghatti, Gum Tragacanth, Hydrogen Peroxide Modified Lecithin, Hydrogenated Isomaltulose, Hydrogenated Poly-1-Decene, Hydrogenated Rapeseed Oil, Hydrogenated Soybean Oil, Hydrogenated Tallow, Hydroxylated Lecithin, Hydroxylated Soybean Oil Fatty Acids, Hydroxypropyl Alginate, Hydroxypropyl Distarch Glycerol, Hydroxypropyl Distarch Phosphate, Hydroxypropyl Starch, Invertase, Isomalt, Isomaltitol, Karaya Gum, Konjac Flour, Lactic And Fatty Acid Esters Of Glycerol, Lactitol, Lactose, Lactylated Fatty Acid Esters Of Glycerol And Propylene Glycol, Lactylic Esters Of Fatty Acids, Lecithin, Levulose, Locust Bean Gum, Low Erucic Acid Rapeseed Oil, Magnesium Caprate, Magnesium Caprylate, Magnesium Dihydrogen Phosphate, Magnesium Hydrogen Phosphate, Magnesium Laurate, Magnesium Myristate, Magnesium Oleate, Magnesium Palmitate, Magnesium Phosphate, Magnesium Salts Of Fatty Acids, Magnesium Stearate, Magnesium Sulfate, Magnesium Sulphate, Maltitol, Maltodextrin, Mannitol, Metatartaric Acid, Methyl Erithrytol Ester Of Colophane, Methyl Erithrytol Esters Of Colophane, Methyl Esters Of Fatty Acids, Methyl Glucoside- Coconut Oil Ester, Methylphenylpolysiloxane, Microcrystalline Wax, Milled Rice, Mineral Oil, Modified Food Starch, Modified Pectin, Modified Starch, Modified Wheat Starch, Mono And Diglycerides Of Fatty Acids, Mono And Di-Glycerides Of Fatty Acids, Mono- And Di-Glycerides Of Fatty Acids, Monoammonium Glutamate, Monoammonium L-Glutamate, Monocalcium Orthophosphate, Monoglyceride Citrate, Monomagnesium Orthophosphate, Monosodium Glutamate, Monosodium L-Glutamate, Monosodium Phosphate Derivatives Of Mono- And Diglycerides, Monostarch Phosphate, Msg, Mustard Flour, Nitrogen, Nitrous Oxide, Oat Gum, Octenyl Succinic Acid, Osa, Otassium Acid Pyrophosphate, Oxidized Hydroxypropyl Starch, Oxidized Starch, Paprika, Partially Hydrogenated Low Erucic Acid Rapeseed Oil, Partially Hydrogenated Low Erucic Acid Rapeseed Oil, Partially Hydrogenated Low Erucic Acid Rapeseed Oil, Partially Hydrolysed Lecithin, Pectin, Peg, Penta Erithrytol Ester Of Colophane, Penta Erithrytol Esters Of Colophane, Pentapotassium Triphosphate, Pentasodium Triphosphate, Peptone, Pes, Petrolatum, Petroleum Jelly, Petroleum Wax, Phenyl Salicylate, Phosphated Distarch Phosphate, Polydextrose, Polydimethylsiloxane, Polydimethylsiloxanev, Polyethylene Glycol, Polyglycerol Esters Of Fatty Acids, Polyglycerol Esters Of Interesterified Ricinoleic Acid, Polyglycerol Polyricinoleate, Polyoxyethylene 20 Sorbitan, Polyoxyethylene 40 Stearate, Polyoxyethylene 8 Stearate, Polysorbate, Polyvinyl Alcohol, Polyvinylpyrrolidone, Potassium Acid Pyrophosphate, Potassium Acid Tartrate, Potassium Alginate, Potassium Caprate, Potassium Caprylate, Potassium Chloride, Potassium Citrate, Potassium Dihydrogen Citrate, Potassium Dihydrogen Phosphate, Potassium Hydroxide, Potassium Lactate, Potassium Laurate, Potassium Myristate, Potassium Oleate, Potassium Palmitate, Potassium Pectinate, Potassium Permanganate, Potassium Phosphate, Potassium Polymetaphosphate, Potassium Polyphosphate, Potassium Salt Of Carrageenan, Potassium Salt Of Furcelleran, Potassium Salts Of Fatty Acids, Potassium Sodium Dextro-Tartrate, Potassium Sodium Tartrate, Potassium Stearate, Potato Starch, Pregelatinized Starch, Processed Euchema Seaweed, Propylene Glycol, Propylene Oxide Copolymer, Protease, Pullulan, Pva, Pva-Peg, Pvoh, Pvp, Quillaia, Rennet, Rice Starch, Salts Of Carrageenan, Salts Of Furcelleran, Salts Of Myristic Palmitic And Stearic Acids With Ammonia Calcium Potassium And Sodium, Salts Of Oleic Acid With Calcium Potassium And Sodium, Shellac, Silicon Dioxide, Sodium Acid Pyrophosphate, Sodium Alginate, Sodium Aluminium Phosphate, Sodium Aluminum Phosphate, Sodium Bicarbonate, Sodium Calcium Alginate, Sodium Calcium Polyphosphate, Sodium Caprate, Sodium Caprylate, Sodium Carbonate, Sodium Carboxymethyl Cellulose, Sodium Carboxymethylcellulose, Sodium Caseinate, Sodium Chloride, Sodium Citrate, Sodium Dextro-Tartrate, Sodium Dihydrogen Citrate, Sodium Dihydrogen Phosphate, Sodium Fluoride, Sodium Gluconate, Sodium Glutamate, Sodium Hydrogen Carbonate, Sodium Lactate, Sodium Lactylate, Sodium Laurate, Sodium Lauryl Sulfate, Sodium Lauryl Sulphate, Sodium Laurylsulfate, Sodium Laurylsulphate, Sodium Metaphosphate, Sodium Myristate, Sodium Oleate, Sodium Oleyl Lactylate, Sodium Palmitate, Sodium Phosphate, Sodium Polyphosphate, Sodium Potassium Hexametaphosphate, Sodium Potassium Tartrate, Sodium Potassium Triphosphate, Sodium Pyrophosphate, Sodium Salt Of Carboxymethyl Cellulose, Sodium Salt Of Carrageenan, Sodium Salt Of Furcelleran, Sodium Salts Of Fatty Acids, Sodium Stearate, Sodium Stearoyl Fumarate, Sodium Stearoyl Lactylate, Sodium Stearoyl-2 Lactylate, Sodium Tartrate, Sodium Thiosulfate, Sodium Thiosulphate, Sodium Tripolyphosphate, Sorbitan Monolaurate, Sorbitan Monooleate, Sorbitan Monopalmitate, Sorbitan Monostearate, Sorbitan Tristearate, Sorbitol, Sorbitol, Soy Protein Isolate, Soybean Hemicellulose, Spermaceti Wax, Starch Acetate, Starch Aluminium Octenyl Succinate, Starch Phosphate, Starch Sodium Octenyl Succinate, Starch Sodium Succinate, Stearic Acid, Stearyl Citrate, Stearyl Monoglyceridyl Citrate, Succinyl Distarch Glycerol, Succinylated Monoglycerides, Succistearin, Sucroglycerides, Sucrose Acetate Isobutyrate, Sucrose Esters Of Fatty Acids, Sucrose Fatty Acid Esters, Sucrose Oligoesters, Superglycerinated Hydrogenated Rapeseed Oil, Synthetic Glycerin, Talc, Tamarind Seed Polysaccharide, Tannic Acid, Tannins, Tapioca Starch, Tara Gum, Tartaric Acid Esters Of Mono- And Di-Glycerides Of Fatty Acids, Tetrapotassium Diphosphate, Tetrasodium Diphosphate, Thermally Oxidized Soya Bean Oil With Mono- And Di-Glycerides Of Fatty Acids, Thickener, Tragacanth Gum, Triacetin, Tricalcium Citrate, Tricalcium Phosphate, Triethyl Citrate, Trimagnesium Phosphate, Triphosphates, Tripotassium Citrate, Tripotassium Phosphate, Trisodium Citrate, Trisodium Diphosphate, Trisodium Phosphate, Unmodified Starch, Unspecified Protein Hydrolysate, Vegetable Gum, Wax Esters, Waxy Cornstarch, Wheat Gluten, Wheat Starch, Whey, Xanthan Gum, Xylitol, Yellow Mustard Extract |

# Supplementary Table 3. Summary of classification criteria for ultra-processing, the Chilean Nutrient Profile Model (NPM), and nutrient thresholds of foods and beverages “high in”

| **Ultra-processed foods** | Products with presence of markers of ultra-processing (e.g., cosmetic additives, aromas, ultra-processed ingredients such as invert sugar, maltodextrins, etc.) in the ingredient list. | |
| --- | --- | --- |
| **Chilean Nutrient Profile Model (NPM)** | Packaged foods and beverages with added sugar, added sodium, or added saturated fat. | |
|  | **Solids (per 100 g)** | **Liquids (per 100 mL)** |
| High in energy | > 275 kcal | > 70 kcal |
| High in sodium | > 400 mg | > 100 mg |
| High in saturated fats | > 4 g | > 3 g |
| High in sugars | > 10 g | > 5 g |

**Supplementary Table 4.** Prevalence of ultra-processed foods and beverages^1^ across North and Latin American markets, Mintel, 2018-2023

|  | N = total products | % UPF | p-value |
| --- | --- | --- | --- |
| United States | 81,693 (39.4) | 81 | Ref. |
|  |  |  |  |
| Argentina | 12,545 (6.1) | 81 | 1.000 |
| Brazil | 42,435 (20.5) | 78 | < 0.001 |
| Chile | 4,998 (2.4) | 81 | 1.000 |
| Colombia | 16,999 (8.2) | 83 | < 0.001 |
| Costa Rica | 4,369 (2.1) | 85 | < 0.001 |
| Ecuador | 5,814 (2.8) | 78 | 0.001 |
| Mexico | 25,169 (12.1) | 82 | < 0.001 |
| Peru | 5,826 (2.8) | 79 | 0.595 |
| Puerto Rico | 4,485 (2.2) | 81 | 1.000 |
| Venezuela | 3,030 (1.5) | 69 | < 0.001 |

^1^ Ultra-processed foods were identified by the presence of markers of ultra-processing, which contain certain ingredients and food additives in the ingredient list.

**Supplementary Table 5.** Percentage of ultra-processed foods in the packaged food supply and estimated 95% confidence intervals, by food categories, across North and Latin America, Mintel, 2018-2023

|  | US | AR | BR | CL | CO | CR | EC | MX | PE | PR | VE |
| --- | --- | --- | --- | --- | --- | --- | --- | --- | --- | --- | --- |
| Baby food | 71  (68, 74) | **89**  **(79, 99)** | **80**  **(76, 85)** | 69  (55, 84) | 75  (69, 82) | 72  (61, 83) | 76  (65, 87) | **87**  **(84, 90)** | 69  (59, 80) | 75  (56, 94) | 88  (73, 100) |
| Bakery products | 93  (92, 93) | **94**  **(93, 95)** | **80**  **(79, 81)** | 93  (91, 95) | **91**  **(90, 92)** | 92  (90, 94) | **88**  **(86, 90)** | 92  (91, 93) | 91  (89, 93) | **97**  **(96, 98)** | **77**  **(73, 80)** |
| Breakfast cereals | 81  (79, 83) | 75  (70, 80) | **61**  **(58, 65)** | **90**  **(85, 95)** | 78  (74, 82) | 86  (80, 91) | 85  (80, 91) | 84  (82, 87) | **66**  **(61, 71)** | 80  (74, 85) | **61**  **(50, 73)** |
| Candy, gum, and chocolate | 98  (98, 99) | 98  (96, 99) | **95**  **(94, 95)** | 99  (98, 100) | 99  (98, 99) | 98  (97, 99) | 98  (96, 100) | 98  (98, 99) | 97  (96, 98) | 98  (97, 100) | 98  (96, 100) |
| Cheese and yogurt | 76  (75, 77) | **98**  **(97, 99)** | **96**  **(95, 97)** | **98**  **(97, 99)** | **93**  **(91, 94)** | **91**  **(88, 95)** | **96**  **(94, 98)** | **92**  **(91, 94)** | **99**  **(97, 100)** | **83**  **(79, 87)** | 83  (74, 92) |
| Desserts and ice cream | 97  (96, 97) | **99**  **(98, 100)** | **94**  **(93, 95)** | **100** | 98  (97, 99) | **99**  **(97, 100)** | 94  (90, 98) | 97  (95, 98) | **100** | 98  (95, 100) | 98  (94, 100) |
| Fruits and vegetables | 42  (40, 43) | 44  (40, 48) | **48**  **(46, 51)** | 39  (32, 46) | **56**  **(52, 60)** | **55**  **(48, 63)** | **56**  **(49, 63)** | 40  (37, 44) | **64**  **(56, 72)** | 35  (29, 41) | **55**  **(49, 62)** |
| Other dairy products | 82  (79, 84) | 77  (69, 86) | **93**  **(91, 95)** | 85  (77, 95) | **92**  **(88, 95)** | 90  (82, 98) | 78  (69, 86) | **96**  **(94, 98)** | **92**  **(86, 98)** | 79  (70, 87) | 78  (68, 88) |
| Pasta, rice, and other starchy dishes | 61  (59, 62) | 59  (55, 62) | **57**  **(54, 59)** | 54  (47, 60) | **45**  **(41, 49)** | 63  (56, 70) | **42**  **(36, 47)** | 58  (55, 61) | 57  (52, 63) | **72**  **(67, 77)** | **25**  **(20, 31)** |
| Processed fish, meat, and egg products | 72  (71, 73) | 69  (65, 72) | **77**  **(75, 79)** | 71  (66, 75) | 75  (73, 78) | 76  (72, 80) | 69  (66, 72) | 73  (71, 74) | **58**  **(54, 63)** | 70  (65, 75) | **31**  **(25, 38)** |
| Ready-to-eat/heat foods | 96  (96, 97) | **85**  **(81, 89)** | **62**  **(60, 64)** | **83**  **(77, 90)** | **78**  **(74, 82)** | **88**  **(81, 94)** | **79**  **(73, 85)** | **89**  **(87, 92)** | **86**  **(80, 92)** | 97  (94, 100) | 81  (66, 96) |
| Sauces and seasonings | 79  (78, 80) | 81  (79, 83) | **72**  **(71, 73)** | **73**  **(70, 77)** | **87**  **(85, 89)** | **87**  **(85, 89)** | **83**  **(80, 87)** | 80  (79, 82) | **88**  **(85, 91)** | 77  (72, 82) | **74**  **(70, 77)** |
| Savory spreads | 80  (78, 82) | 77  (69, 85) | 77  (71, 83) | 90  (81, 99) | 87  (80, 93) | **93**  **(85, 100)** | 91  (82, 99) | 87  (82, 92) | **95**  **(88, 100)** | 68  (54, 83) | 75  (62, 88) |
| Snacks (savory and sweet) | 76  (76, 77) | **62**  **(59, 65)** | **62**  **(61, 65)** | **68**  **(64, 72)** | **64**  **(62, 66)** | **71**  **(67, 75)** | **51**  **(46, 56)** | **71**  **(70, 73)** | **56**  **(52, 60)** | **71**  **(67, 75)** | **52**  **(45, 59)** |
| Sweet spreads | 60  (58, 63) | **78**  **(74, 81)** | **77**  **(75, 80)** | **84**  **(78, 91)** | **89**  **(86, 92)** | **87**  **(80, 94)** | **79**  **(72, 86)** | **82**  **(79, 85)** | **86**  **(80, 91)** | **89**  **(80, 97)** | **93**  **(89, 97)** |
| Total | 79  (78, 79) | **81**  **(80, 82)** | **77**  **(76, 77)** | **81**  **(80, 83)** | **82**  **(81, 83)** | **84**  **(82, 85)** | **77**  **(75, 78)** | **81**  **(81, 82)** | 78  (77, 79) | **80**  **(78, 81)** | **67**  **(65, 69)** |

US: United States; AR: Argentina; BR: Brazil; CL: Chile; CO: Colombia; CR: Costa Rica; EC: Ecuador; MX: Mexico; PE: Peru; PR: Puerto Rico; VE: Venezuela

Estimates were compared between the U.S. and other countries within food/beverage group; bolded estimates are significantly different (p < 0.05).

# Supplementary Table 6. Percentage of ultra-processed beverages in the packaged food supply and estimated 95% confidence intervals, by beverage categories, across North and Latin America, Mintel, 2018-2023

|  | US | AR | BR | CL | CO | CR | EC | MX | PE | PR | VE |
| --- | --- | --- | --- | --- | --- | --- | --- | --- | --- | --- | --- |
| Beverage mixes and concentrates | 92  (90, 94) | **99**  **(97, 100)** | **99**  **(98, 100)** | 94  (89, 100) | 96  (94, 98) | **97**  **(94, 100)** | 90  (83, 97) | 94  (92, 97) | 85  (77, 92) | 96  (91, 100) | 81  (70, 91) |
| Carbonated soft drinks | 98  (97, 99) | 99  (97, 100) | 99  (98, 100) | **100** | 100  (99, 100) | **100** | **100** | 96  (94, 98) | **100** | 99  (97, 100) | 97  (90, 100) |
| Coffee, tea, and hot chocolate | 81  (79, 83) | **57**  **(52, 62)** | **87**  **(85, 89)** | 75  (68, 81) | 78  (75, 82) | 81  (74, 89) | 83  (78, 88) | 80  (77, 83) | **56**  **(50, 63)** | 88  (80, 96) | **65**  **(57, 73)** |
| Juice and fruit drinks | 73  (70, 75) | **86**  **(83, 90)** | **65**  **(63, 68)** | 70  (64, 76) | **87**  **(84, 90)** | **88**  **(83, 92)** | 79  (73, 84) | 77  (74, 80) | **96**  **(94, 99)** | **84**  **(80, 88)** | **97**  **(94, 100)** |
| Milk, other dairy beverages, and plant-based alternatives | 63  (60, 66) | **78**  **(74, 82)** | **91**  **(89, 92)** | **80**  **(73, 86)** | **88**  **(86, 90)** | **91**  **(86, 96)** | **81**  **(78, 85)** | **77**  **(74, 79)** | **92**  **(89, 95)** | 59  (51, 68) | 71  (58, 85) |
| Meal replacements and nutritional drinks | 89  (88, 91) | **72**  **(61, 82)** | 88  (86, 89) | 84  (68, 100) | 90  (86, 95) | 71  (49, 92) | 84  (73, 96) | 93  (91, 95) | **98**  **(93, 100)** | 93  (79, 100) | **100** |
| Plain and flavored water | 87  (85, 89) | 90  (81, 98) | 82  (70, 94) | 77  (62, 92) | **73**  **(66, 81)** | 82  (71, 93) | 88  (77, 98) | **80**  **(73, 82)** | **63**  **(41, 85)** | 80  (71, 89) | 91  (74, 100) |
| Sports and energy drinks | 100  (99, 100) | 100 | 99  (98, 100) | 100 | 100 | 100 | 100 | 99  (99, 100) | 100 | 100 | 100 |
| Total | 89  (89, 90) | **82**  **(80, 83)** | **81**  **(80, 82)** | **80**  **(78, 83)** | **87**  **(86, 88)** | 89  (87, 92) | **84**  **(82, 86)** | **85**  **(83, 87)** | **85**  **(83, 87)** | **86**  **(83, 88)** | **82**  **(79, 86)** |

US: United States; AR: Argentina; BR: Brazil; CL: Chile; CO: Colombia; CR: Costa Rica; EC: Ecuador; MX: Mexico; PE: Peru; PR: Puerto Rico; VE: Venezuela

Estimates were compared between the U.S. and other countries within food/beverage group; bolded estimates are significantly different (p < 0.05).

**Supplementary Table 7.** Prevalence of foods high in energy, sugar, sodium, or saturated fat in the packaged food supply across North and Latin America, Mintel, 2018-2023

|  | Energy | | | Sugar | | | Sodium | | | Saturated fats | | | Any nutrient^2^ | | |
| --- | --- | --- | --- | --- | --- | --- | --- | --- | --- | --- | --- | --- | --- | --- | --- |
|  | UPF | Non-UPF | p^1^ | UPF | Non-UPF | p^1^ | UPF | Non-UPF | p^1^ | UPF | Non-UPF | p^1^ | UPF | Non-UPF | p^1^ |
| United States | 65 | 58 | (ref.) | 49 | 22 | (ref.) | 46 | 46 | (ref.) | 45 | 38 | (ref.) | 87 | 73 | (ref.) |
|  |  |  |  |  |  |  |  |  |  |  |  |  |  |  |  |
| Argentina | **72** | **55** | 0.005 | 56 | 24 | 0.222 | 35 | 34 | 0.589 | 44 | 30 | 0.054 | **88** | **63** | 0.001 |
| Brazil | **72** | **55** | < 0.001 | 52 | 22 | 0.393 | **24** | **34** | < 0.001 | **57** | **34** | < 0.001 | 85 | 70 | 0.736 |
| Chile | 66 | 62 | 0.536 | **44** | **25** | 0.046 | 27 | 29 | 0.588 | **51** | **27** | < 0.001 | 77 | 63 | 0.113 |
| Colombia | **64** | **53** | 0.013 | 47 | 20 | 0.476 | **38** | **32** | < 0.001 | **50** | **34** | < 0.001 | **84** | **63** | 0.002 |
| Costa Rica | 68 | 58 | 0.500 | 52 | 19 | 0.074 | **33** | **43** | 0.006 | 51 | 40 | 0.379 | 84 | 72 | 0.190 |
| Ecuador | 63 | 53 | 0.221 | 43 | 20 | 0.502 | **46** | **38** | 0.001 | **51** | **34** | 0.001 | **89** | **71** | 0.024 |
| Mexico | 64 | 58 | 0.440 | **50** | **26** | 0.006 | 37 | 36 | 0.440 | **45** | **34** | < 0.001 | 84 | 70 | 0.230 |
| Peru | 69 | 62 | 0.801 | 52 | 24 | 0.822 | **26** | **19** | 0.003 | **43** | **27** | < 0.001 | 77 | 55 | 0.500 |
| Puerto Rico | **69** | **52** | 0.002 | **51** | **17** | 0.007 | **46** | **35** | < 0.001 | 45 | 34 | 0.068 | **87** | **61** | < 0.001 |
| Venezuela | **61** | **31** | < 0.001 | 49 | 16 | 0.072 | 40 | 34 | 0.195 | **31** | **16** | 0.025 | **81** | **51** | 0.015 |

^1^ p-interaction between country vs. USA and UPF vs. non-UPF; ^2^ High in sodium, sugar, or saturated fat.

Bolded estimates are ones for which the p-interaction is < 0.05.

# Supplementary Table 8. Prevalence of beverages high in energy, sugar, sodium, or saturated fat in the packaged food supply across North and Latin America, Mintel, 2018-2023

|  | Energy | | | Sugar | | | Sodium | | | Saturated fats | | | Any nutrient^2^ | | |
| --- | --- | --- | --- | --- | --- | --- | --- | --- | --- | --- | --- | --- | --- | --- | --- |
|  | UPF | Non-UPF | p^1^ | UPF | Non-UPF | p^1^ | UPF | Non-UPF | p^1^ | UPF | Non-UPF | p^1^ | UPF | Non-UPF | p^1^ |
| United States | 21 | 15 | (ref.) | 25 | 28 | (ref.) | 29 | 12 | (ref.) | 17 | 6 | (ref.) | 54 | 37 | (ref.) |
|  |  |  |  |  |  |  |  |  |  |  |  |  |  |  |  |
| Argentina | 37 | 11 | 0.300 | 38 | 22 | 0.248 | 37 | 0 | - | 10 | 0 | - | 61 | 22 | 0.201 |
| Brazil | **52** | **15** | 0.003 | **42** | **26** | 0.015 | **19** | **33** | < 0.001 | **17** | **26** | < 0.001 | **57** | **62** | 0.014 |
| Chile | 21 | 15 | 0.921 | 26 | 23 | 0.448 | 13 | 8 | 0.419 | **9** | **38** | < 0.001 | **36** | **46** | 0.008 |
| Colombia | 31 | 24 | 0.623 | **42** | **33** | 0.005 | 14 | 7 | 0.216 | **11** | **14** | < 0.001 | 52 | 44 | 0.071 |
| Costa Rica | 24 | 21 | 0.676 | **46** | **21** | 0.043 | 13 | 0 | - | **8** | **14** | 0.026 | 57 | 29 | 0.376 |
| Ecuador | 24 | 24 | 0.201 | **43** | **30** | 0.020 | **10** | **18** | < 0.001 | **6** | **8** | 0.007 | **51** | **50** | 0.035 |
| Mexico | **17** | **18** | 0.024 | **40** | **30** | 0.001 | **15** | **17** | < 0.001 | **7** | **10** | < 0.001 | **53** | **49** | 0.004 |
| Peru | 22 | 18 | 0.720 | **33** | **5** | 0.015 | **13** | **18** | 0.008 | **7** | **18** | < 0.001 | 43 | 36 | 0.392 |
| Puerto Rico | 21 | 5 | 0.100 | **52** | **33** | 0.005 | 18 | 2 | 0.320 | 10 | 2 | 0.788 | 67 | 38 | 0.120 |
| Venezuela | 25 | 0 | - | 44 | 33 | 0.610 | 11 | 0 | - | 4 | 0 | - | 53 | 33 | 0.905 |

^1^ p-interaction between country vs. USA and UPF vs. non-UPF; ^2^ High in sodium, sugar, or saturated fat.

Bolded estimates are ones for which the p-interaction is < 0.05.
